# Supplementary figures and images for: Characterization of BLUF-photoreceptors present in Acinetobacter nosocomialis
Source: PLoS One. 2022 Apr 20;17(4):e0254291. doi: 10.1371/journal.pone.0254291 (PMC9020721; doi:10.1371/journal.pone.0254291)

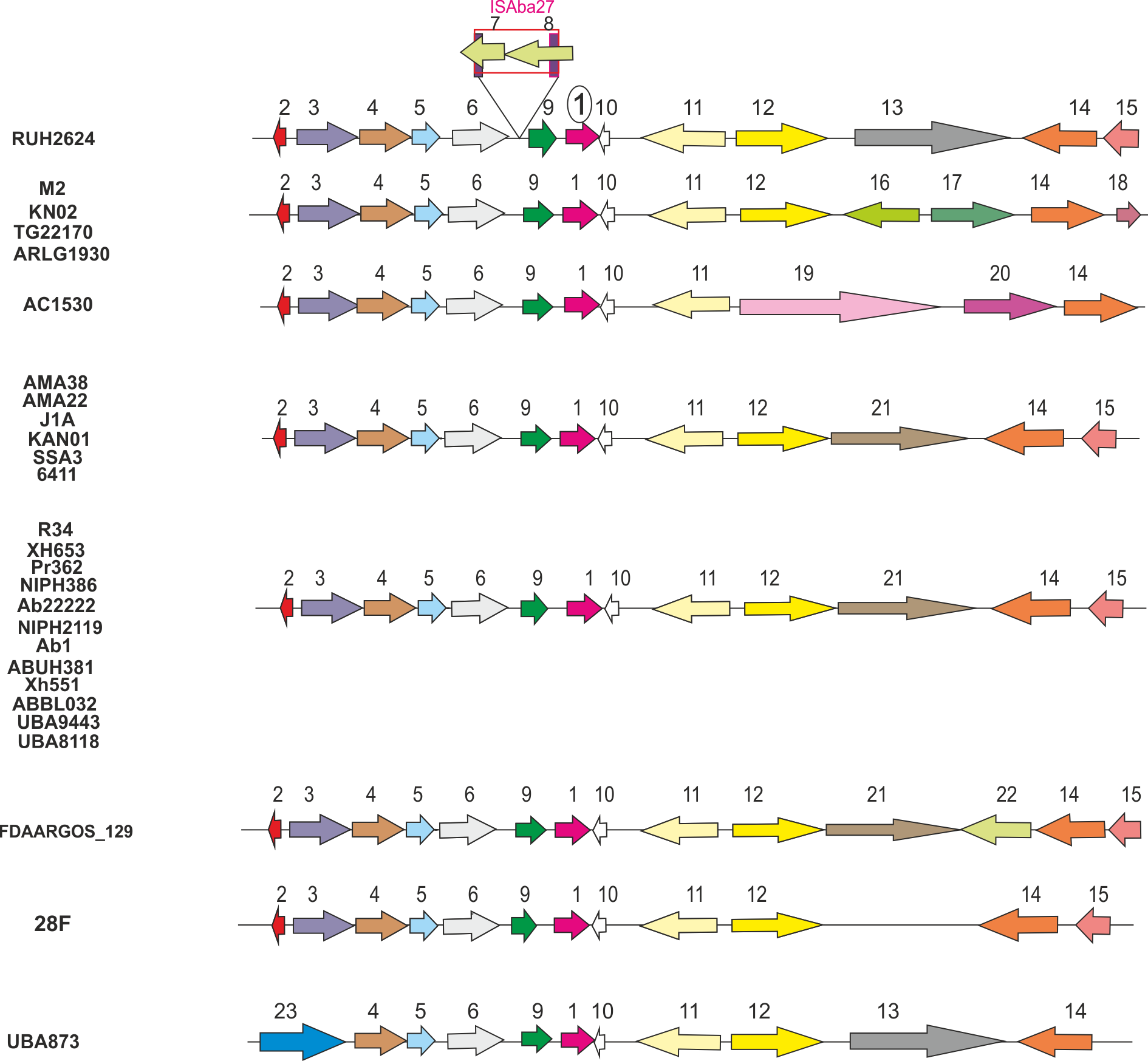

Supplement: S1 Fig — (A). Coding-sequences (CDSs) are located in their corresponding frame. AnBLUF65 is indicated as pink arrow. Different colors indicate different functions. Gene annotations are indicated as numbers above the schemes, with the following codes: 1- AnBLUF65, 2- DUF2171, 3- Acyl-CoA-dehydrogenase, 4- GlcNAc-PI-de-N-acetylase, 5- methyltransferase domain, 6- glycosyltransferase, 7- DDE endonuclease domain, putative transposase, 8- helix-turn-helix fo DDE superfamily endonuclease, 9- BOF- class 2b aminoacyl-tRNA synthetases- NirD/YgiW/Y damily stress tolerance protein, 10- HP, 11- poly (R)-hydroxyalkanoic acid synthase, 12- sodium/glutamate symporter, 13- proton antiporter-2 (CPA2) family, 14- NDAB- Rossmann Superfamily- Oxidoreductase, 15- DoxX-like family, 16- LysR family transcriptional regulator, 17- AKR15A family of aldo-keto reductase, 18- pyrabactin resistance 1 (PYR1) receptor, 19- type I secretion target GGXGXDXXX repeat protein, 20- Paax domain, 21- glutathione-regulated potassium-efflux system protein KefC, 22- IS3 family transposase, 23- L- asparagine transporter. (TIF) [file pone.0254291.s001.tif]

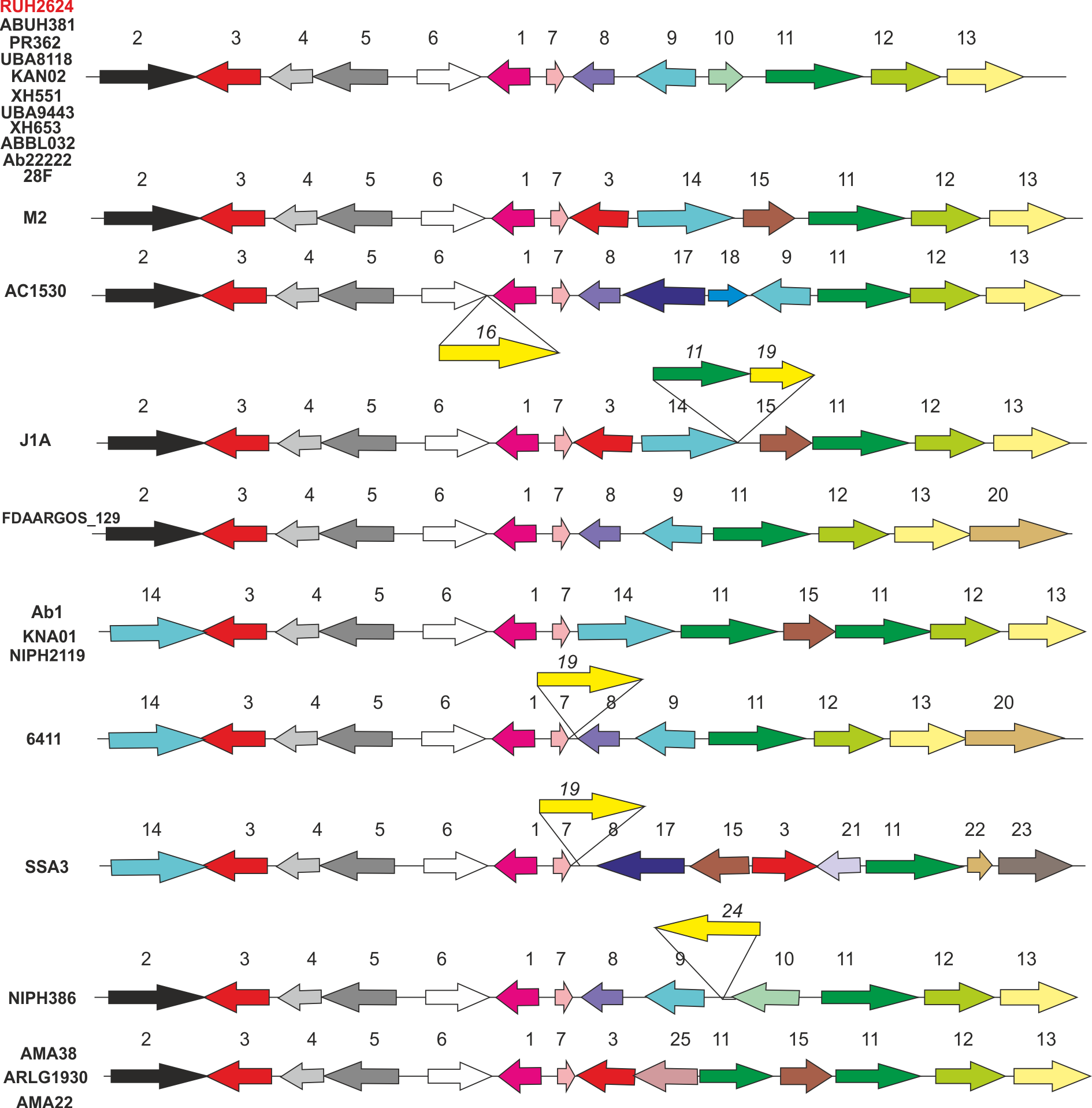

Supplement: S2 Fig — (A). Coding-sequences (CDSs) are located in their corresponding frame. AnBLUF46 is indicated as pink arrow. Different colors indicate different functions. Gene annotations are indicated as numbers above the schemes, with the following codes: 1- AnBLUF46, 2- aromatic acid:H+ symporter (AAHS), 3- AraC family transcriptional regulator RmlC-cupin protein, 4–3-hydroxyburyrate dehydrogenase, 5- H+/gluconate symporter, 6- MBL fold metallo-hydrolase, 7- HP, 8- DUF2846- putative conjugal domain, 9- DUF3298- putative heat shock protein, 10- HP, 11- LysR family transcriptional regulator, 12- succinyl-CoA:3- ketoacid-coenzyme A transferase subunit A, 13- succinyl-CoA:3-ketoacid-coenzyme A transferase subunit B, 14- multidrug efflux MFS transporter, 15- pimeloyl-ACP methyl ester carboxylesterase, 16- IS5 transposase, 17- TetR/AcrR family transcriptional regulator, 18- DoxX family protein, 19- IS transposase, 20- anion permease ArsB/NhaD, 21–3-oxoacyl-ACP reductase FabG, 22- nuclear transport factor 2 family protein, 23- fermentarion-respiration switch protein FrsA, 24- DDE transposase domain, 25- Bcr/CflA family drug resistance efflux transporter. (TIF) [file pone.0254291.s002.tif]

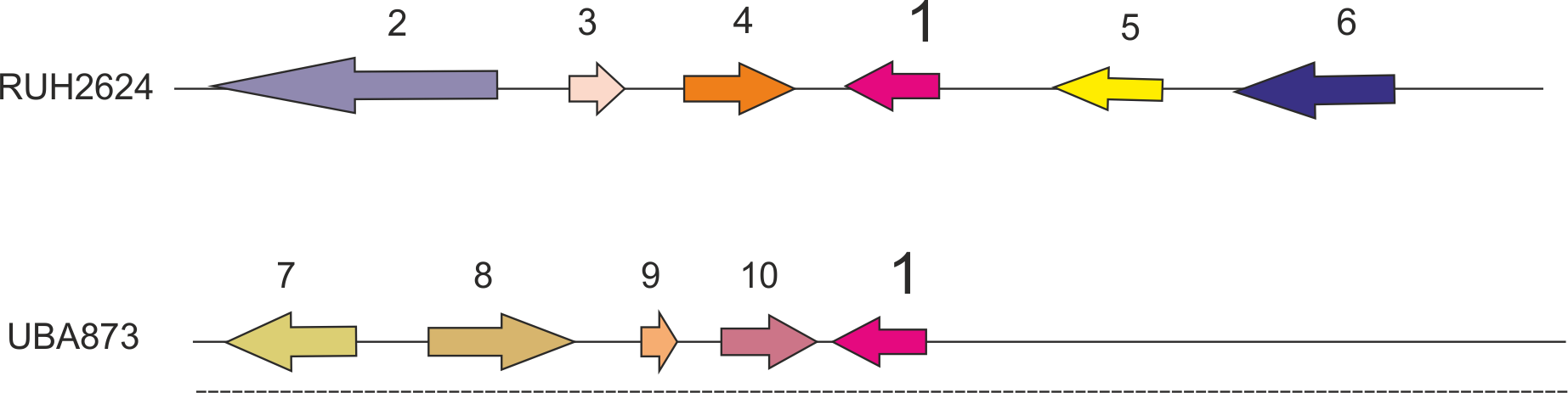

Supplement: S3 Fig — (A). Coding-sequences (CDSs) are located in their corresponding frame. AnBLUF85 is indicated as pink arrow. Different colors indicate different functions. Gene annotations are indicated as numbers above the schemes, with the following codes: 1- AnBLUF85, 2- relaxase-MobA/MobL family protein, 3- FAM199X, 4-HP, 5- N-acetiltransferasa, 6- conjugal transfer pilus assembly protein TraB, 7-RepB initiator replication protein, 8- potasium transporter, 9-mRNA-degrading endonuclease RelE, 10- chromate transport protein ChrA. (TIF) [file pone.0254291.s003.tif]
