# Supplementary material for: Characterization of BLUF-photoreceptors present in Acinetobacter nosocomialis
Source: PLoS One. 2022 Apr 20;17(4):e0254291. doi: 10.1371/journal.pone.0254291 (PMC9020721; doi:10.1371/journal.pone.0254291)
Supplement: S1 Table — (†): Interactions types and subtypes: Van der Waals (VDW), hydrogen bond (HBOND), ππ stackings (PIPISTACK), main chain (MC), side chain (SC) and ligand (LIG) IAC: generic interaction that simply indicates a generic contact based on a distance cutoff. (*): in Å between atom centers / mass centers / barycenters depending on the type of interaction and the type of residue. (‡) Orientation:Parallel (P), Lateral (L), Normal (N), Tilted Edge to Face (T-EF), Tilted Face to Edge (T-FE). (PDF) [file pone.0254291.s004.pdf]

S1 Table: Residue Interaction Network Generator (RING) data for AnBLUF65 and AnBLUF46. (†): Interactions types and subtypes:Van der Waals (VDW), hydrogen bond (HBOND), ππ stackings (PIPISTACK), main chain (MC), side chain (SC) and ligand (LIG) IAC: generic interaction that simply indicates a generic contact based on a distance cutoff. (*): in Å between atom centers / mass centers / barycenters depending on the type of interaction and the type of residue. (‡) Orientation:Parallel (P), Lateral (L), Normal (N), Tilted Edge to Face (T-EF), Tilted Face to Edge (T-FE)

NodeId1	Interaction (†)	NodeId2	Distance(*)	Angle	Energy	Atom1	Atom2	Donor	Positive	Cation	Orientation (‡)
**AnBLUF46 DARK**											
A:2:_:SER	VDW:MC_SC	A:57:_:GLU	3.971	-999.9	6	C	CD				
A:2:_:SER	HBOND:MC_MC	A:58:_:LYS	2.938	32.011	17	O	N	A:58:_:LYS			
A:3:_:LEU	VDW:SC_MC	A:56:_:GLY	3.407	-999.9	6	CD2	C				
A:3:_:LEU	HBOND:MC_MC	A:86:_:SER	2.888	9.342	17	O	N	A:86:_:SER			
A:3:_:LEU	VDW:SC_SC	A:89:_:ARG	3.977	-999.9	6	CD1	CD				
A:4:_:ILE	HBOND:MC_MC	A:56:_:GLY	2.881	8.174	17	N	O	A:4:_:ILE			
A:4:_:ILE	VDW:SC_SC	A:58:_:LYS	3.891	-999.9	6	CD1	CG				
A:4:_:ILE	VDW:SC_SC	A:61:_:VAL	3.835	-999.9	6	CD1	CG1				
A:4:_:ILE	VDW:SC_SC	A:83:_:LEU	3.567	-999.9	6	CD1	CD1				
A:5:_:GLY	VDW:MC_SC	A:53:_:TYR	3.682	-999.9	6	C	CE2				
A:5:_:GLY	HBOND:MC_MC	A:84:_:GLU	2.843	8.095	17	N	O	A:5:_:GLY			
A:6:_:PHE	VDW:MC_SC	A:53:_:TYR	3.724	-999.9	6	C	CD2				
A:6:_:PHE	HBOND:MC_MC	A:54:_:LEU	2.862	12.813	17	N	O	A:6:_:PHE			
A:6:_:PHE	VDW:SC_SC	A:61:_:VAL	3.663	-999.9	6	CB	CG1				
A:6:_:PHE	PIPISTACK:SC_SC	A:65:_:PHE	4.928	137.753	9.4	-10.069,-22.918,11.220	-9.751,-18.855,13.991				T-EF n2.67,p3.65
A:6:_:PHE	VDW:SC_SC	A:65:_:PHE	3.118	-999.9	6	CE1	CE1				
A:6:_:PHE	VDW:SC_SC	A:80:_:ILE	3.478	-999.9	6	CZ	CG2				
A:6:_:PHE	VDW:SC_SC	A:83:_:LEU	3.66	-999.9	6	CD2	CB				
A:7:_:MET	VDW:SC_SC	A:51:_:LEU	3.794	-999.9	6	CE	CD2				
A:7:_:MET	VDW:SC_SC	A:53:_:TYR	3.78	-999.9	6	CE	CB				
A:7:_:MET	HBOND:MC_MC	A:81:_:ILE	2.906	16.183	17	O	N	A:81:_:ILE			
A:7:_:MET	VDW:SC_SC	A:81:_:ILE	3.904	-999.9	6	CG	CD1				
A:7:_:MET	HBOND:MC_MC	A:82:_:PHE	3.066	9.74	17	N	O	A:7:_:MET			
A:7:_:MET	VDW:SC_SC	A:82:_:PHE	3.433	-999.9	6	CB	CE2				
A:7:_:MET	VDW:SC_SC	A:127:_:ILE	3.568	-999.9	6	SD	CG2				
A:7:_:MET	VDW:SC_SC	A:131:_:ILE	3.583	-999.9	6	CE	CG1				
A:8:_:TYR	HBOND:MC_MC	A:52:_:GLN	2.984	12.564	17	O	N	A:52:_:GLN			
A:8:_:TYR	VDW:SC_SC	A:52:_:GLN	3.28	-999.9	6	CE2	OE1				
A:8:_:TYR	PIPISTACK:SC_SC	A:65:_:PHE	3.89	17.902	9.4	-13.395,-18.556,15.320	-9.751,-18.855,13.991				P n3.39,p1.79
A:8:_:TYR	VDW:SC_SC	A:65:_:PHE	3.151	-999.9	6	CE2	CD2				
A:8:_:TYR	VDW:SC_SC	A:69:_:ILE	3.914	-999.9	6	CZ	CG2				
A:8:_:TYR	PIPISTACK:SC_SC	A:75:_:HIS	5.998	109.521	9.4	-13.395,-18.556,15.320	-12.073,-14.027,19.023				N n2.43,p5.17
A:8:_:TYR	VDW:SC_SC	A:75:_:HIS	3.816	-999.9	6	CE1	CE1				
A:8:_:TYR	VDW:SC_SC	A:78:_:CYS	3.249	-999.9	6	CD1	SG				
A:8:_:TYR	VDW:MC_SC	A:81:_:ILE	3.962	-999.9	6	C	CD1				
A:9:_:ALA	HBOND:MC_MC	A:79:_:GLU	2.815	4.427	17	O	N	A:79:_:GLU			
A:9:_:ALA	VDW:SC_MC	A:119:_:PRO	3.826	-999.9	6	CB	C				
A:10:_:SER	VDW:MC_SC	A:49:_:TYR	3.696	-999.9	6	C	CD2				
A:10:_:SER	HBOND:MC_MC	A:50:_:PHE	2.889	10.782	17	O	N	A:50:_:PHE			
A:10:_:SER	VDW:SC_SC	A:75:_:HIS	4.006	-999.9	6	CB	CG				
A:11:_:LYS	VDW:SC_SC	A:49:_:TYR	3.445	-999.9	6	CB	CE2				
A:11:_:LYS	HBOND:MC_MC	A:76:_:GLN	2.717	17.581	17	O	N	A:76:_:GLN			
A:11:_:LYS	VDW:SC_SC	A:77:_:ASN	3.956	-999.9	6	CB	CB				
A:12:_:THR	HBOND:MC_MC	A:48:_:GLY	2.826	23.258	17	N	O	A:12:_:THR			
A:12:_:THR	VDW:SC_MC	A:48:_:GLY	3.592	-999.9	6	CB	C				
A:12:_:THR	VDW:SC_SC	A:50:_:PHE	3.996	-999.9	6	CB	CE2				
A:13:_:ASN	HBOND:MC_MC	A:16:_:HIS	3.361	15.936	17	O	N	A:16:_:HIS			
A:13:_:ASN	VDW:SC_SC	A:16:_:HIS	3.633	-999.9	6	ND2	CD2				
A:13:_:ASN	HBOND:MC_MC	A:74:_:ARG	2.957	9.953	17	N	O	A:13:_:ASN			
A:13:_:ASN	VDW:SC_MC	A:74:_:ARG	3.63	-999.9	6	CB	C				
A:16:_:HIS	HBOND:SC_MC	A:73:_:SER	3.113	25.322	17	NE2	O	A:16:_:HIS			
A:18:_:GLN	HBOND:MC_MC	A:21:_:GLN	3.281	46.916	17	O	N	A:21:_:GLN			
A:18:_:GLN	HBOND:MC_MC	A:22:_:ASP	3.214	6.029	17	O	N	A:22:_:ASP			
A:19:_:ILE	HBOND:MC_MC	A:22:_:ASP	3.27	60.454	17	O	N	A:22:_:ASP			
A:19:_:ILE	HBOND:MC_MC	A:23:_:LEU	2.931	7.73	17	O	N	A:23:_:LEU			
A:19:_:ILE	VDW:SC_SC	A:23:_:LEU	3.664	-999.9	6	CG2	CD1				
A:20:_:LYS	HBOND:MC_MC	A:24:_:ILE	3.191	10.978	17	O	N	A:24:_:ILE			
A:20:_:LYS	VDW:SC_SC	A:24:_:ILE	4.004	-999.9	6	CG	CD1				
A:21:_:GLN	HBOND:MC_MC	A:24:_:ILE	3.199	56.756	17	O	N	A:24:_:ILE			
A:21:_:GLN	HBOND:MC_MC	A:25:_:ASP	2.797	4.08	17	O	N	A:25:_:ASP			
A:22:_:ASP	HBOND:MC_MC	A:25:_:ASP	3.392	60.542	17	O	N	A:25:_:ASP			
A:22:_:ASP	HBOND:MC_MC	A:26:_:ILE	2.873	9.109	17	O	N	A:26:_:ILE			
A:22:_:ASP	VDW:SC_SC	A:74:_:ARG	3.767	-999.9	6	CG	CD				
A:23:_:LEU	HBOND:MC_MC	A:27:_:LEU	3.148	10.588	17	O	N	A:27:_:LEU			
A:23:_:LEU	VDW:SC_SC	A:45:_:TYR	4.002	-999.9	6	CD2	CG				
A:23:_:LEU	VDW:SC_SC	A:50:_:PHE	3.623	-999.9	6	CD2	CE1				
A:23:_:LEU	VDW:SC_SC	A:98:_:PHE	3.918	-999.9	6	CD2	CZ				
A:24:_:ILE	HBOND:MC_MC	A:27:_:LEU	3.347	54.344	17	O	N	A:27:_:LEU			
A:24:_:ILE	HBOND:MC_MC	A:28:_:THR	2.938	8.889	17	O	N	A:28:_:THR			
A:25:_:ASP	HBOND:MC_MC	A:28:_:THR	3.168	57.581	17	O	N	A:28:_:THR			
A:25:_:ASP	HBOND:MC_MC	A:29:_:GLU	2.898	5.311	17	O	N	A:29:_:GLU			
A:25:_:ASP	VDW:MC_SC	A:29:_:GLU	4.03	-999.9	6	C	CD				
A:26:_:ILE	HBOND:MC_MC	A:30:_:ALA	2.9	13.017	17	O	N	A:30:_:ALA			
A:26:_:ILE	VDW:SC_SC	A:43:_:LEU	3.755	-999.9	6	CG2	CD2				
A:26:_:ILE	VDW:SC_SC	A:50:_:PHE	3.935	-999.9	6	CD1	CZ				
A:26:_:ILE	VDW:SC_SC	A:74:_:ARG	3.716	-999.9	6	CG1	CZ				
A:27:_:LEU	HBOND:MC_MC	A:30:_:ALA	3.403	58.826	17	O	N	A:30:_:ALA			
A:27:_:LEU	HBOND:MC_MC	A:31:_:VAL	2.894	7.292	17	O	N	A:31:_:VAL			
A:27:_:LEU	VDW:SC_SC	A:98:_:PHE	3.558	-999.9	6	CD1	CG				
A:27:_:LEU	VDW:SC_SC	A:144:_:GLN	3.285	-999.9	6	CD1	NE2				
A:28:_:THR	HBOND:MC_MC	A:32:_:LYS	3.345	10.668	17	O	N	A:32:_:LYS			
A:29:_:GLU	HBOND:MC_MC	A:33:_:PHE	3.007	12.247	17	O	N	A:33:_:PHE			
A:29:_:GLU	IONIC:SC_SC	A:74:_:ARG	3.456	139.995	20	-12.614,-4.660,13.657	CZ		A:74:_:ARG		
A:29:_:GLU	VDW:SC_SC	A:74:_:ARG	3.876	-999.9	6	CD	CZ				
A:30:_:ALA	HBOND:MC_MC	A:34:_:ASN	2.854	14.189	17	O	N	A:34:_:ASN			
A:30:_:ALA	VDW:MC_SC	A:34:_:ASN	3.945	-999.9	6	C	CG				
A:30:_:ALA	VDW:SC_SC	A:96:_:MET	3.93	-999.9	6	CB	CB				
A:31:_:VAL	HBOND:MC_MC	A:35:_:SER	3.188	17.003	17	O	N	A:35:_:SER			
A:31:_:VAL	VDW:SC_SC	A:96:_:MET	4.019	-999.9	6	CG2	CB				
A:32:_:LYS	HBOND:MC_SC	A:35:_:SER	3.132	53.755	17	O	OG	A:35:_:SER			
A:32:_:LYS	HBOND:MC_MC	A:36:_:GLN	3.399	15.349	17	O	N	A:36:_:GLN			
A:33:_:PHE	HBOND:MC_MC	A:36:_:GLN	3.277	50.534	17	O	N	A:36:_:GLN			
A:33:_:PHE	HBOND:MC_MC	A:37:_:ASN	3.053	37.31	17	O	N	A:37:_:ASN			
A:33:_:PHE	VDW:SC_SC	A:37:_:ASN	3.496	-999.9	6	CE2	OD1				
A:33:_:PHE	VDW:SC_SC	A:60:_:GLN	3.356	-999.9	6	CZ	NE2				
A:33:_:PHE	VDW:SC_SC	A:64:_:LEU	3.672	-999.9	6	CE1	CD1				
A:34:_:ASN	HBOND:MC_MC	A:37:_:ASN	3.372	29.726	17	O	N	A:37:_:ASN			
A:34:_:ASN	HBOND:MC_MC	A:38:_:ASP	3.088	56.02	17	O	N	A:38:_:ASP			
A:34:_:ASN	HBOND:MC_MC	A:39:_:ILE	2.879	3.402	17	O	N	A:39:_:ILE			
A:34:_:ASN	VDW:SC_SC	A:39:_:ILE	3.71	-999.9	6	CG	CG2				
A:34:_:ASN	VDW:SC_SC	A:64:_:LEU	3.435	-999.9	6	OD1	CD2				
A:34:_:ASN	VDW:SC_SC	A:96:_:MET	3.266	-999.9	6	ND2	CE				
A:37:_:ASN	VDW:SC_SC	A:60:_:GLN	3.446	-999.9	6	ND2	CD				
A:39:_:ILE	VDW:SC_SC	A:60:_:GLN	3.648	-999.9	6	CD1	CG				
A:39:_:ILE	VDW:SC_SC	A:64:_:LEU	3.544	-999.9	6	CD1	CD2				
A:40:_:THR	HBOND:MC_MC	A:55:_:GLU	2.803	9.044	17	O	N	A:55:_:GLU			
A:40:_:THR	HBOND:SC_SC	A:89:_:ARG	2.769	52.569	17	OG1	NH1	A:89:_:ARG			
A:40:_:THR	VDW:SC_MC	A:91:_:PHE	3.858	-999.9	6	CB	C				
A:41:_:GLY	VDW:MC_SC	A:91:_:PHE	3.904	-999.9	6	C	CB				
A:41:_:GLY	HBOND:MC_MC	A:94:_:TRP	3.049	27.288	17	N	O	A:41:_:GLY			
A:41:_:GLY	VDW:MC_SC	A:96:_:MET	3.724	-999.9	6	C	SD				
A:41:_:GLY	HBOND:MC_MC	A:97:_:LYS	2.853	6.93	17	O	N	A:97:_:LYS			

A:42:_:VAL	HBOND:MC_MC	A:53:_:TYR	2.97	8.935	17	O	N	A:53:_:TYR	
A:42:_:VAL	VDW:SC_SC	A:53:_:TYR	3.992	-999.9	6	CG1	CB		
A:42:_:VAL	VDW:SC_SC	A:91:_:PHE	3.429	-999.9	6	CG1	CE1		
A:42:_:VAL	VDW:MC_SC	A:96:_:MET	3.419	-999.9	6	C	SD		
A:42:_:VAL	VDW:SC_SC	A:97:_:LYS	3.814	-999.9	6	CG2	CB		
A:42:_:VAL	VDW:SC_SC	A:134:_:LEU	3.787	-999.9	6	CG1	CD1		
A:43:_:LEU	VDW:SC_SC	A:50:_:PHE	3.794	-999.9	6	CD1	CD1		
A:43:_:LEU	VDW:SC_SC	A:52:_:GLN	3.775	-999.9	6	CD1	CB		
A:43:_:LEU	VDW:SC_SC	A:96:_:MET	3.899	-999.9	6	CB	SD		
A:43:_:LEU	HBOND:MC_MC	A:97:_:LYS	2.712	6.283	17	N	O	A:43:_:LEU	
A:43:_:LEU	VDW:SC_SC	A:98:_:PHE	3.864	-999.9	6	CD2	CE2		
A:43:_:LEU	HBOND:MC_MC	A:99:_:ALA	2.915	22.298	17	O	N	A:99:_:ALA	
A:44:_:TYR	HBOND:MC_MC	A:51:_:LEU	2.744	2.159	17	N	O	A:44:_:TYR	
A:44:_:TYR	VDW:SC_MC	A:99:_:ALA	4.021	-999.9	6	CD1	C		
A:44:_:TYR	VDW:SC_MC	A:100:_:PRO	3.597	-999.9	6	CE1	C		
A:44:_:TYR	VDW:SC_SC	A:105:_:ILE	3.915	-999.9	6	CE1	CD1		
A:44:_:TYR	VDW:SC_SC	A:119:_:PRO	3.843	-999.9	6	CE2	CG		
A:44:_:TYR	PIPISTACK:SC_SC	A:130:_:PHE	5.171	89.053	9.4	-25.316,-18.643,17.843	-24.945,-23.372,15.786		N n0.33,p5.59
A:44:_:TYR	VDW:SC_SC	A:130:_:PHE	3.764	-999.9	6	CD2	CZ		
A:44:_:TYR	VDW:SC_SC	A:134:_:LEU	3.853	-999.9	6	CB	CD2		
A:45:_:TYR	HBOND:SC_MC	A:48:_:GLY	3.393	42.847	17	OH	N	A:48:_:GLY	
A:45:_:TYR	VDW:SC_MC	A:48:_:GLY	3.826	-999.9	6	CE1	C		
A:45:_:TYR	PIPISTACK:SC_SC	A:50:_:PHE	5.282	125.241	9.4	-22.863,-12.154,21.581	-18.160,-12.961,19.315		T-EF n1.55,p5.61
A:45:_:TYR	VDW:SC_SC	A:50:_:PHE	3.615	-999.9	6	CD1	CE1		
A:45:_:TYR	VDW:SC_SC	A:98:_:PHE	3.815	-999.9	6	CB	CZ		
A:45:_:TYR	HBOND:MC_MC	A:99:_:ALA	3.042	16.195	17	N	O	A:45:_:TYR	
A:46:_:GLY	HBOND:MC_MC	A:49:_:TYR	3.067	10.234	17	N	O	A:46:_:GLY	
A:47:_:ASN	HBOND:SC_SC	A:116:_:GLU	2.643	38.925	17	ND2	OE1	A:47:_:ASN	
A:47:_:ASN	VDW:SC_SC	A:116:_:GLU	3.549	-999.9	6	ND2	CB		
A:47:_:ASN	VDW:SC_SC	A:118:_:ASN	3.638	-999.9	6	ND2	CB		
A:49:_:TYR	VDW:SC_SC	A:118:_:ASN	3.089	-999.9	6	CD2	OD1		
A:49:_:TYR	VDW:SC_SC	A:119:_:PRO	3.826	-999.9	6	CB	CD		
A:50:_:PHE	PIPISTACK:SC_SC	A:75:_:HIS	6.187	98.743	9.4	-18.160,-12.961,19.315	-12.073,-14.027,19.023		N n3.34,p3.20
A:50:_:PHE	VDW:MC_SC	A:119:_:PRO	3.535	-999.9	6	C	CG		
A:51:_:LEU	VDW:SC_SC	A:119:_:PRO	3.63	-999.9	6	CB	CB		
A:51:_:LEU	VDW:SC_SC	A:122:_:LEU	3.426	-999.9	6	CD1	CD1		
A:51:_:LEU	VDW:SC_SC	A:130:_:PHE	3.628	-999.9	6	CD1	CE2		
A:51:_:LEU	VDW:SC_SC	A:134:_:LEU	3.804	-999.9	6	CD2	CD1		
A:52:_:GLN	VDW:SC_SC	A:69:_:ILE	3.459	-999.9	6	OE1	CD1		
A:52:_:GLN	VDW:SC_SC	A:96:_:MET	3.377	-999.9	6	NE2	CE		
A:53:_:TYR	PIPISTACK:SC_SC	A:82:_:PHE	5.891	120.211	9.4	-18.086,-22.936,7.263	-15.873,-27.570,10.151		T-FE n1.98,p1.52
A:53:_:TYR	VDW:SC_SC	A:82:_:PHE	3.534	-999.9	6	CE2	CZ		
A:53:_:TYR	PIPISTACK:SC_SC	A:91:_:PHE	5.619	115.45	9.4	-18.086,-22.936,7.263	-22.959,-20.377,6.133		N n2.75,p2.00
A:53:_:TYR	VDW:SC_SC	A:91:_:PHE	3.493	-999.9	6	CD1	CD2		
A:54:_:LEU	VDW:SC_SC	A:64:_:LEU	3.997	-999.9	6	CD2	CD2		
A:54:_:LEU	VDW:SC_SC	A:65:_:PHE	3.496	-999.9	6	CD1	CB		
A:54:_:LEU	VDW:SC_SC	A:96:_:MET	3.789	-999.9	6	CD2	CE		
A:55:_:GLU	VDW:MC_SC	A:61:_:VAL	3.909	-999.9	6	C	CG2		
A:55:_:GLU	HBOND:SC_SC	A:89:_:ARG	2.936	10.175	17	OE1	NE	A:89:_:ARG	
A:55:_:GLU	VDW:SC_MC	A:89:_:ARG	4.013	-999.9	6	CD	C		
A:55:_:GLU	HBOND:SC_MC	A:90:_:LEU	2.837	51.828	17	OE2	N	A:90:_:LEU	
A:55:_:GLU	HBOND:SC_MC	A:91:_:PHE	2.951	20.853	17	OE2	N	A:91:_:PHE	
A:55:_:GLU	VDW:SC_SC	A:91:_:PHE	3.971	-999.9	6	CD	CB		
A:56:_:GLY	VDW:MC_SC	A:61:_:VAL	3.675	-999.9	6	C	CG2		
A:57:_:GLU	HBOND:MC_MC	A:60:_:GLN	3.169	48.92	17	O	N	A:60:_:GLN	
A:57:_:GLU	HBOND:MC_MC	A:61:_:VAL	2.906	5.632	17	O	N	A:61:_:VAL	
A:58:_:LYS	HBOND:MC_MC	A:61:_:VAL	3.426	56.771	17	O	N	A:61:_:VAL	
A:58:_:LYS	HBOND:MC_MC	A:62:_:GLU	2.931	6.75	17	O	N	A:62:_:GLU	
A:58:_:LYS	VDW:SC_SC	A:62:_:GLU	3.584	-999.9	6	CE	CD		
A:59:_:GLU	HBOND:MC_MC	A:63:_:THR	2.897	15.946	17	O	N	A:63:_:THR	
A:60:_:GLN	HBOND:MC_SC	A:63:_:THR	3.23	22.701	17	O	OG1	A:63:_:THR	
A:60:_:GLN	HBOND:MC_MC	A:64:_:LEU	2.964	22.182	17	O	N	A:64:_:LEU	
A:61:_:VAL	HBOND:MC_MC	A:64:_:LEU	3.242	47.46	17	O	N	A:64:_:LEU	
A:61:_:VAL	HBOND:MC_MC	A:65:_:PHE	2.703	7.48	17	O	N	A:65:_:PHE	
A:62:_:GLU	VDW:MC_MC	A:65:_:PHE	3.738	-999.9	6	C	C		
A:62:_:GLU	HBOND:MC_MC	A:67:_:LYS	3.257	16.974	17	O	N	A:67:_:LYS	
A:62:_:GLU	VDW:SC_SC	A:83:_:LEU	3.968	-999.9	6	CD	CD1		
A:63:_:THR	VDW:SC_SC	A:67:_:LYS	3.824	-999.9	6	CB	CD		
A:63:_:THR	HBOND:MC_SC	A:68:_:SER	3.344	56.008	17	O	OG	A:68:_:SER	
A:64:_:LEU	HBOND:MC_MC	A:68:_:SER	3.032	33.215	17	O	N	A:68:_:SER	
A:64:_:LEU	HBOND:MC_MC	A:69:_:ILE	3	7.24	17	O	N	A:69:_:ILE	
A:64:_:LEU	VDW:SC_SC	A:69:_:ILE	3.863	-999.9	6	CG	CD1		
A:65:_:PHE	VDW:SC_SC	A:69:_:ILE	3.574	-999.9	6	CD2	CG2		
A:65:_:PHE	VDW:SC_SC	A:78:_:CYS	3.396	-999.9	6	CZ	SG		
A:65:_:PHE	VDW:SC_SC	A:80:_:ILE	3.842	-999.9	6	CE1	CG1		
A:66:_:TYR	HBOND:MC_MC	A:70:_:LEU	3.468	28.114	17	O	N	A:70:_:LEU	
A:66:_:TYR	VDW:MC_SC	A:70:_:LEU	3.926	-999.9	6	C	CB		
A:68:_:SER	HBOND:MC_MC	A:71:_:LYS	3.167	19.52	17	O	N	A:71:_:LYS	
A:69:_:ILE	HBOND:MC_MC	A:72:_:ASP	2.987	10.178	17	O	N	A:72:_:ASP	
A:69:_:ILE	HBOND:MC_SC	A:75:_:HIS	2.726	2.889	17	O	NE2	A:75:_:HIS	
A:69:_:ILE	VDW:SC_SC	A:75:_:HIS	3.403	-999.9	6	CG2	CE1		
A:70:_:LEU	VDW:SC_SC	A:78:_:CYS	3.473	-999.9	6	CD1	CB		
A:72:_:ASP	VDW:SC_SC	A:75:_:HIS	3.922	-999.9	6	CB	CD2		
A:77:_:ASN	VDW:SC_SC	A:120:_:TYR	3.719	-999.9	6	ND2	CE2		
A:79:_:GLU	VDW:SC_SC	A:120:_:TYR	3.81	-999.9	6	CB	CD2		
A:82:_:PHE	VDW:SC_SC	A:127:_:ILE	3.53	-999.9	6	CE2	CG1		
A:89:_:ARG	HBOND:MC_MC	A:92:_:LYS	3.36	12.702	17	O	N	A:92:_:LYS	
A:90:_:LEU	VDW:SC_SC	A:131:_:ILE	3.875	-999.9	6	CD1	CG2		
A:91:_:PHE	HBOND:MC_MC	A:94:_:TRP	2.922	15.487	17	O	N	A:94:_:TRP	
A:91:_:PHE	VDW:SC_SC	A:94:_:TRP	3.871	-999.9	6	CD1	CZ3		
A:91:_:PHE	VDW:SC_SC	A:131:_:ILE	3.697	-999.9	6	CE2	CG2		
A:91:_:PHE	VDW:SC_SC	A:135:_:VAL	3.311	-999.9	6	CZ	CG2		
A:94:_:TRP	VDW:SC_SC	A:97:_:LYS	3.548	-999.9	6	CG	CB		
A:97:_:LYS	HBOND:SC_MC	A:134:_:LEU	2.702	29.568	17	NZ	O	A:97:_:LYS	
A:97:_:LYS	HBOND:SC_MC	A:135:_:VAL	2.695	60.278	17	NZ	O	A:97:_:LYS	
A:97:_:LYS	HBOND:SC_MC	A:137:_:GLN	2.717	20.573	17	NZ	O	A:97:_:LYS	
A:97:_:LYS	VDW:SC_SC	A:139:_:ASN	3.331	-999.9	6	CG	ND2		
A:98:_:PHE	HBOND:MC_MC	A:140:_:LEU	3.034	10.564	17	O	N	A:140:_:LEU	
A:98:_:PHE	VDW:SC_SC	A:140:_:LEU	3.585	-999.9	6	CE1	CD2		
A:99:_:ALA	VDW:SC_SC	A:134:_:LEU	3.998	-999.9	6	CB	CD2		
A:99:_:ALA	VDW:SC_SC	A:137:_:GLN	3.519	-999.9	6	CB	OE1		
A:100:_:PRO	VDW:SC_SC	A:137:_:GLN	3.267	-999.9	6	CD	NE2		
A:100:_:PRO	VDW:SC_SC	A:140:_:LEU	3.426	-999.9	6	CD	CB		
A:102:_:ASN	HBOND:SC_MC	A:105:_:ILE	2.755	4.194	17	OD1	N	A:105:_:ILE	
A:102:_:ASN	VDW:SC_SC	A:105:_:ILE	3.403	-999.9	6	OD1	CG1		
A:102:_:ASN	HBOND:MC_MC	A:106:_:LYS	3.012	10.83	17	O	N	A:106:_:LYS	
A:102:_:ASN	HBOND:SC_SC	A:137:_:GLN	3.215	20.024	17	ND2	OE1	A:102:_:ASN	
A:102:_:ASN	VDW:SC_SC	A:137:_:GLN	3.033	-999.9	6	ND2	CD		
A:103:_:THR	HBOND:MC_MC	A:106:_:LYS	3.199	59.506	17	O	N	A:106:_:LYS	
A:103:_:THR	HBOND:MC_MC	A:107:_:ASP	2.844	8.207	17	O	N	A:107:_:ASP	
A:104:_:LYS	HBOND:MC_MC	A:108:_:PHE	3.061	15.105	17	O	N	A:108:_:PHE	
A:104:_:LYS	VDW:SC_SC	A:133:_:LEU	3.91	-999.9	6	CB	CD2		
A:105:_:ILE	HBOND:MC_MC	A:108:_:PHE	3.224	54.623	17	O	N	A:108:_:PHE	
A:105:_:ILE	HBOND:MC_MC	A:109:_:PHE	3.03	14.686	17	O	N	A:109:_:PHE	
A:105:_:ILE	VDW:SC_SC	A:109:_:PHE	3.794	-999.9	6	CG2	CD2		
A:105:_:ILE	VDW:SC_SC	A:130:_:PHE	3.852	-999.9	6	CG2	CE1		
A:105:_:ILE	VDW:SC_SC	A:137:_:GLN	3.026	-999.9	6	CD1	OE1		

A:106:_:LYS	HBOND:MC_MC	A:109:_:PHE	3.425	48.753	17	O	N	A:109:_:PHE	
A:106:_:LYS	HBOND:MC_MC	A:110:_:PHE	3	8.656	17	O	N	A:110:_:PHE	
A:106:_:LYS	VDW:SC_SC	A:117:_:PHE	3.647	-999.9	6	CG	CE1		
A:107:_:ASP	HBOND:MC_MC	A:111:_:HIS	2.903	13.703	17	O	N	A:111:_:HIS	
A:108:_:PHE	HBOND:MC_MC	A:112:_:HIS	3.399	21.24	17	O	N	A:112:_:HIS	
A:108:_:PHE	PIPISTACK:SC_SC	A:112:_:HIS	4.898	60.634	9.4	-28.372,-28.409,17.148	-29.203,-32.743,19.273		N n3.33,p1.72
A:108:_:PHE	VDW:SC_SC	A:112:_:HIS	3.436	-999.9	6	CE1	CE1		
A:108:_:PHE	VDW:SC_SC	A:122:_:LEU	3.856	-999.9	6	CE2	CD2		
A:108:_:PHE	VDW:SC_SC	A:126:_:SER	3.986	-999.9	6	CZ	CB		
A:108:_:PHE	VDW:SC_SC	A:129:_:SER	3.425	-999.9	6	CE1	CB		
A:108:_:PHE	PIPISTACK:SC_SC	A:130:_:PHE	6.243	155.305	9.4	-28.372,-28.409,17.148	-24.945,-23.372,15.786		P n3.32,p5.47
A:108:_:PHE	VDW:SC_SC	A:133:_:LEU	3.623	-999.9	6	CB	CD1		
A:109:_:PHE	HBOND:MC_MC	A:112:_:HIS	3.491	38.825	17	O	N	A:112:_:HIS	
A:109:_:PHE	HBOND:MC_MC	A:113:_:HIS	3.422	59.885	17	O	N	A:113:_:HIS	
A:109:_:PHE	HBOND:MC_MC	A:114:_:VAL	2.896	17.573	17	O	N	A:114:_:VAL	
A:109:_:PHE	VDW:SC_SC	A:114:_:VAL	3.886	-999.9	6	CD1	CG1		
A:109:_:PHE	PIPISTACK:SC_SC	A:117:_:PHE	5.943	80.995	9.4	-26.617,-24.518,21.322	-30.343,-21.442,24.783		N n1.81,p6.60
A:109:_:PHE	VDW:SC_SC	A:117:_:PHE	3.627	-999.9	6	CB	CD1		
A:109:_:PHE	VDW:SC_SC	A:122:_:LEU	3.86	-999.9	6	CZ	CD2		
A:109:_:PHE	PIPISTACK:SC_SC	A:130:_:PHE	5.895	94.853	9.4	-26.617,-24.518,21.322	-24.945,-23.372,15.786		N n2.22,p5.54
A:109:_:PHE	VDW:SC_SC	A:130:_:PHE	3.79	-999.9	6	CE2	CE1		
A:110:_:PHE	HBOND:MC_MC	A:113:_:HIS	3.076	15.299	17	O	N	A:113:_:HIS	
A:114:_:VAL	VDW:SC_SC	A:121:_:LEU	3.295	-999.9	6	CG1	CD1		
A:118:_:ASN	HBOND:MC_MC	A:121:_:LEU	3.257	11.675	17	O	N	A:121:_:LEU	
A:118:_:ASN	VDW:SC_SC	A:121:_:LEU	3.993	-999.9	6	CB	CD1		
A:119:_:PRO	HBOND:MC_MC	A:122:_:LEU	3.166	14.527	17	O	N	A:122:_:LEU	
A:119:_:PRO	VDW:SC_SC	A:122:_:LEU	4.039	-999.9	6	CB	CD1		
A:122:_:LEU	VDW:SC_SC	A:126:_:SER	3.799	-999.9	6	CD2	CB		
A:122:_:LEU	VDW:SC_SC	A:130:_:PHE	3.422	-999.9	6	CD1	CD2		
A:123:_:ASN	HBOND:SC_MC	A:126:_:SER	3.088	47.991	17	OD1	N	A:126:_:SER	
A:124:_:THR	HBOND:MC_MC	A:127:_:ILE	3.347	27.015	17	O	N	A:127:_:ILE	
A:124:_:THR	VDW:MC_SC	A:127:_:ILE	3.946	-999.9	6	C	CG2		
A:125:_:ASN	VDW:MC_SC	A:128:_:PRO	3.799	-999.9	6	C	CD		
A:126:_:SER	HBOND:MC_MC	A:129:_:SER	3.296	51.807	17	O	N	A:129:_:SER	
A:126:_:SER	HBOND:MC_MC	A:130:_:PHE	2.93	15.05	17	O	N	A:130:_:PHE	
A:127:_:ILE	HBOND:MC_MC	A:130:_:PHE	3.449	52.584	17	O	N	A:130:_:PHE	
A:127:_:ILE	HBOND:MC_MC	A:131:_:ILE	2.935	10.354	17	O	N	A:131:_:ILE	
A:127:_:ILE	VDW:SC_SC	A:131:_:ILE	3.467	-999.9	6	CD1	CD1		
A:128:_:PRO	HBOND:MC_MC	A:132:_:GLU	3.416	11.622	17	O	N	A:132:_:GLU	
A:129:_:SER	HBOND:MC_MC	A:132:_:GLU	3.333	59.389	17	O	N	A:132:_:GLU	
A:129:_:SER	HBOND:MC_MC	A:133:_:LEU	2.911	14.906	17	O	N	A:133:_:LEU	
A:130:_:PHE	HBOND:MC_MC	A:133:_:LEU	3.323	51.204	17	O	N	A:133:_:LEU	
A:130:_:PHE	HBOND:MC_MC	A:134:_:LEU	3.036	10.313	17	O	N	A:134:_:LEU	
A:130:_:PHE	VDW:SC_SC	A:134:_:LEU	3.826	-999.9	6	CE2	CD1		
A:131:_:ILE	HBOND:MC_MC	A:134:_:LEU	3.106	55.003	17	O	N	A:134:_:LEU	
A:131:_:ILE	HBOND:MC_MC	A:135:_:VAL	2.835	13.288	17	O	N	A:135:_:VAL	
A:132:_:GLU	HBOND:MC_MC	A:135:_:VAL	3.281	54.005	17	O	N	A:135:_:VAL	
A:132:_:GLU	HBOND:MC_MC	A:136:_:ASP	2.998	14.986	17	O	N	A:136:_:ASP	
A:133:_:LEU	HBOND:MC_MC	A:136:_:ASP	3.483	48.297	17	O	N	A:136:_:ASP	
A:133:_:LEU	HBOND:MC_MC	A:137:_:GLN	2.952	35.214	17	O	N	A:137:_:GLN	
**LIGAND INTERACTIONS**									
_:1:_:FMN	IAC:LIG_SC	A:8:_:TYR	2.955	-999.9	0	C7M	HH		
_:1:_:FMN	IAC:LIG_SC	A:10:_:SER	4.838	-999.9	0	C7M	OG		
_:1:_:FMN	IAC:LIG_SC	A:12:_:THR	6.218	-999.9	0	C7M	HG1		
_:1:_:FMN	IAC:LIG_SC	A:13:_:ASN	6.88	-999.9	0	C8M	H		
_:1:_:FMN	IAC:LIG_SC	A:22:_:ASP	6.517	-999.9	0	C8M	HB3		
_:1:_:FMN	IAC:LIG_SC	A:23:_:LEU	6.574	-999.9	0	C7M	HD22		
_:1:_:FMN	IAC:LIG_MC	A:25:_:ASP	6.611	-999.9	0	C1'	O		
_:1:_:FMN	VDW:LIG_SC	A:26:_:ILE	3.749	-999.9	6	C6	CG2		
_:1:_:FMN	IAC:LIG_SC	A:27:_:LEU	6.303	-999.9	0	N5	HD22		
_:1:_:FMN	IAC:LIG_SC	A:29:_:GLU	2.638	-999.9	0	O3'	OE1		
_:1:_:FMN	VDW:LIG_SC	A:30:_:ALA	3.363	-999.9	6	C4	CB		
_:1:_:FMN	IAC:LIG_MC	A:31:_:VAL	5.492	-999.9	0	N3	N		
_:1:_:FMN	IAC:LIG_MC	A:32:_:LYS	6.108	-999.9	0	O2	C		
_:1:_:FMN	IAC:LIG_SC	A:33:_:PHE	2.245	-999.9	0	O2	HB3		
_:1:_:FMN	IAC:LIG_SC	A:34:_:ASN	2.256	-999.9	0	O4	HD22		
_:1:_:FMN	IAC:LIG_SC	A:37:_:ASN	6.671	-999.9	0	O2	HB2		
_:1:_:FMN	IAC:LIG_SC	A:39:_:ILE	4.522	-999.9	0	O4	HG21		
_:1:_:FMN	IAC:LIG_MC	A:40:_:THR	6.749	-999.9	0	O4	O		
_:1:_:FMN	IAC:LIG_SC	A:41:_:GLY	6.388	-999.9	0	O4	HA1		
_:1:_:FMN	IAC:LIG_SC	A:43:_:LEU	3.189	-999.9	0	C7M	HD11		
_:1:_:FMN	IAC:LIG_MC	A:44:_:TYR	6.91	-999.9	0	C7M	O		
_:1:_:FMN	IAC:LIG_SC	A:45:_:TYR	6.784	-999.9	0	C7M	HD1		
_:1:_:FMN	IAC:LIG_SC	A:50:_:PHE	3.844	-999.9	0	C7M	CG		
_:1:_:FMN	IAC:LIG_MC	A:51:_:LEU	6.709	-999.9	0	C7M	N		
_:1:_:FMN	VDW:LIG_SC	A:52:_:GLN	3.747	-999.9	6	C6	CD		
_:1:_:FMN	IAC:LIG_MC	A:53:_:TYR	6.486	-999.9	0	O4	O		
_:1:_:FMN	IAC:LIG_SC	A:54:_:LEU	2.933	-999.9	0	O4	HD23		
_:1:_:FMN	IAC:LIG_SC	0	6.773	-999.9	0	O2	HE22		
_:1:_:FMN	VDW:LIG_SC	A:64:_:LEU	3.548	-999.9	6	C2	CD1		
_:1:_:FMN	IAC:LIG_SC	A:65:_:PHE	5.923	-999.9	0	C6	HD2		
_:1:_:FMN	IAC:LIG_SC	A:68:_:SER	4.015	-999.9	0	O4'	HB3		
_:1:_:FMN	VDW:LIG_SC	A:69:_:ILE	3.869	-999.9	6	C4	CD1		
_:1:_:FMN	IAC:LIG_MC	A:70:_:LEU	5.737	-999.9	0	O2'	N		
_:1:_:FMN	IAC:LIG_SC	A:71:_:LYS	5.702	-999.9	0	O2'	H		
_:1:_:FMN	IAC:LIG_SC	A:72:_:ASP	2.597	-999.9	0	O2'	HB2		
_:1:_:FMN	IAC:LIG_SC	A:73:_:SER	6.078	-999.9	0	O2'	H		
_:1:_:FMN	IAC:LIG_SC	A:74:_:ARG	2.428	-999.9	0	O3'	HH22		
_:1:_:FMN	IAC:LIG_SC	A:75:_:HIS	3.753	-999.9	0	C7M	ND1		
_:1:_:FMN	IAC:LIG_MC	A:95:_:SER	5.442	-999.9	0	O4	O		
_:1:_:FMN	IAC:LIG_SC	A:96:_:MET	2.672	-999.9	0	O4	HE1		
_:1:_:FMN	IAC:LIG_SC	A:98:_:PHE	6.897	-999.9	0	C7M	HE2		

NodeId1	Interaction	NodeId2	Distance(*)	Angle	Energy	Atom1	Atom2	Donor	Positive	Cation	Orientation
**AnBLUF65 DARK**											
A:1:_:MET	VDW:MC_SC	A:56:_:GLU	3.963	-999.9	6	C	CD				
A:1:_:MET	HBOND:MC_MC	A:57:_:LYS	2.881	32.401	17	O	N	A:57:_:LYS			
A:1:_:MET	VDW:SC_SC	A:83:_:SER	3.843	-999.9	6	SD	CB				
A:2:_:ASN	VDW:SC_MC	A:55:_:GLY	3.661	-999.9	6	OD1	C				
A:2:_:ASN	HBOND:MC_MC	A:84:_:ILE	3.155	18.244	17	O	N	A:84:_:ILE			
A:2:_:ASN	VDW:SC_SC	A:84:_:ILE	3.293	-999.9	6	CB	CG2				
A:3:_:VAL	HBOND:MC_MC	A:55:_:GLY	2.812	11.563	17	N	O	A:3:_:VAL			
A:3:_:VAL	VDW:SC_SC	A:57:_:LYS	3.807	-999.9	6	CG1	CB				
A:3:_:VAL	VDW:SC_SC	A:60:_:VAL	4.037	-999.9	6	CG2	CG1				
A:3:_:VAL	VDW:SC_SC	A:81:_:THR	3.992	-999.9	6	CG2	CG2				
A:4:_:ARG	VDW:SC_SC	A:52:_:CYS	3.487	-999.9	6	CD	SG				
A:4:_:ARG	HBOND:SC_SC	A:54:_:GLU	2.689	24.839	17	NE	OE2	A:4:_:ARG			
A:4:_:ARG	HBOND:MC_MC	A:82:_:TYR	2.777	8.871	17	N	O	A:4:_:ARG			
A:4:_:ARG	VDW:SC_SC	A:82:_:TYR	3.905	-999.9	6	CD	CZ				
A:4:_:ARG	VDW:SC_SC	A:84:_:ILE	3.939	-999.9	6	CZ	CD1				
A:4:_:ARG	HBOND:SC_MC	A:86:_:GLU	2.825	60.265	17	NH2	O	A:4:_:ARG			
A:4:_:ARG	HBOND:SC_SC	A:88:_:SER	3.196	20.464	17	NH2	OG	A:4:_:ARG			
A:4:_:ARG	VDW:SC_SC	A:129:_:LEU	4.02	-999.9	6	CD	CD1				
A:5:_:LEU	HBOND:MC_MC	A:53:_:LEU	2.846	9.315	17	N	O	A:5:_:LEU			
A:5:_:LEU	VDW:SC_SC	A:60:_:VAL	3.673	-999.9	6	CB	CG1				
A:5:_:LEU	VDW:SC_SC	A:64:_:PHE	3.716	-999.9	6	CD1	CD2				
A:6:_:CYS	VDW:SC_SC	A:50:_:PHE	3.78	-999.9	6	CB	CZ				
A:6:_:CYS	VDW:SC_SC	A:52:_:CYS	3.867	-999.9	6	SG	CB				
A:6:_:CYS	HBOND:MC_MC	A:79:_:LEU	2.938	17.951	17	O	N	A:79:_:LEU			
A:6:_:CYS	HBOND:MC_MC	A:80:_:CYS	2.963	7.715	17	N	O	A:6:_:CYS			
A:6:_:CYS	VDW:SC_SC	A:80:_:CYS	3.877	-999.9	6	CB	SG				
A:7:_:TYR	HBOND:MC_MC	A:51:_:GLN	2.964	11.953	17	O	N	A:51:_:GLN			
A:7:_:TYR	VDW:SC_SC	A:51:_:GLN	3.307	-999.9	6	CE2	OE1				
A:7:_:TYR	PIPISTACK:SC_SC	A:64:_:PHE	5.217	143.443	9.4	-13.360,-18.553,15.332	-8.682,-20.106,13.622				T-FE n4.15,p2.68
A:7:_:TYR	VDW:SC_SC	A:64:_:PHE	3.473	-999.9	6	CD2	CE1				
A:7:_:TYR	VDW:SC_SC	A:67:_:ILE	3.748	-999.9	6	CZ	CG2				
A:7:_:TYR	PIPISTACK:SC_SC	A:73:_:HIS	5.987	109.798	9.4	-13.360,-18.553,15.332	-12.069,-14.019,19.022				N n1.26,p5.29
A:7:_:TYR	VDW:SC_SC	A:73:_:HIS	3.812	-999.9	6	CE1	CE1				
A:7:_:TYR	VDW:SC_SC	A:76:_:VAL	3.727	-999.9	6	CD1	CG1				
A:8:_:ALA	HBOND:MC_MC	A:77:_:LYS	2.735	5.023	17	O	N	A:77:_:LYS			
A:8:_:ALA	VDW:SC_MC	A:117:_:PRO	3.831	-999.9	6	CB	C				
A:9:_:SER	HBOND:MC_MC	A:49:_:PHE	2.873	9.503	17	O	N	A:49:_:PHE			
A:9:_:SER	VDW:SC_SC	A:73:_:HIS	4.003	-999.9	6	CB	CG				
A:9:_:SER	VDW:MC_SC	A:118:_:ILE	3.878	-999.9	6	C	CD1				
A:10:_:GLN	VDW:SC_SC	A:13:_:GLU	3.926	-999.9	6	NE2	CD				
A:10:_:GLN	VDW:SC_SC	A:47:_:ASN	3.775	-999.9	6	NE2	CB				
A:10:_:GLN	HBOND:MC_MC	A:74:_:HIS	2.853	13.918	17	O	N	A:74:_:HIS			
A:10:_:GLN	HBOND:SC_SC	A:116:_:ASN	3.149	30.634	17	OE1	ND2	A:116:_:ASN			
A:10:_:GLN	VDW:SC_SC	A:118:_:ILE	3.309	-999.9	6	OE1	CD1				
A:11:_:ARG	VDW:SC_SC	A:18:_:LEU	3.299	-999.9	6	CD	CD1				
A:11:_:ARG	HBOND:SC_SC	A:44:_:TYR	3.464	52.42	17	NE	OH	A:11:_:ARG			
A:11:_:ARG	VDW:SC_SC	A:44:_:TYR	3.756	-999.9	6	CD	CE1				
A:11:_:ARG	HBOND:MC_MC	A:47:_:ASN	2.801	14.404	17	N	O	A:11:_:ARG			
A:11:_:ARG	VDW:SC_MC	A:47:_:ASN	3.666	-999.9	6	CB	C				
A:11:_:ARG	VDW:SC_SC	A:49:_:PHE	3.961	-999.9	6	CB	CE2				
A:11:_:ARG	VDW:MC_SC	A:74:_:HIS	3.913	-999.9	6	C	CE1				
A:12:_:ASN	HBOND:MC_MC	A:15:_:ASN	3.258	15.489	17	O	N	A:15:_:ASN			
A:12:_:ASN	HBOND:MC_MC	A:72:_:ARG	2.923	7.425	17	N	O	A:12:_:ASN			
A:12:_:ASN	VDW:SC_MC	A:72:_:ARG	3.676	-999.9	6	CB	C				
A:15:_:ASN	VDW:SC_SC	A:21:_:ASP	3.663	-999.9	6	ND2	CG				
A:15:_:ASN	HBOND:SC_SC	A:71:_:GLN	2.886	42.711	17	ND2	OE1	A:15:_:ASN			
A:15:_:ASN	VDW:SC_SC	A:71:_:GLN	3.552	-999.9	6	CG	NE2				
A:15:_:ASN	VDW:SC_SC	A:72:_:ARG	3.689	-999.9	6	ND2	CG				
A:17:_:ASP	HBOND:SC_MC	A:20:_:GLN	3.056	13.902	17	OD1	N	A:20:_:GLN			
A:17:_:ASP	VDW:SC_SC	A:20:_:GLN	3.606	-999.9	6	CG	CB				
A:17:_:ASP	HBOND:MC_MC	A:21:_:ASP	3.231	7.476	17	O	N	A:21:_:ASP			
A:18:_:LEU	HBOND:MC_MC	A:21:_:ASP	3.245	60.568	17	O	N	A:21:_:ASP			
A:18:_:LEU	HBOND:MC_MC	A:22:_:LEU	2.908	7.518	17	O	N	A:22:_:LEU			
A:18:_:LEU	VDW:SC_SC	A:22:_:LEU	3.631	-999.9	6	CD1	CG				
A:18:_:LEU	VDW:SC_SC	A:44:_:TYR	3.748	-999.9	6	CD2	CE2				
A:19:_:LEU	HBOND:MC_MC	A:23:_:ARG	3.194	10.133	17	O	N	A:23:_:ARG			
A:19:_:LEU	VDW:SC_SC	A:23:_:ARG	3.806	-999.9	6	CD1	CD				
A:20:_:GLN	HBOND:MC_MC	A:23:_:ARG	3.205	55.223	17	O	N	A:23:_:ARG			
A:20:_:GLN	HBOND:MC_MC	A:24:_:ASP	2.832	5.276	17	O	N	A:24:_:ASP			
A:21:_:ASP	HBOND:MC_MC	A:24:_:ASP	3.414	58.967	17	O	N	A:24:_:ASP			
A:21:_:ASP	HBOND:MC_MC	A:25:_:ILE	2.9	9.259	17	O	N	A:25:_:ILE			
A:21:_:ASP	VDW:SC_SC	A:72:_:ARG	3.745	-999.9	6	CG	CD				
A:22:_:LEU	HBOND:MC_MC	A:26:_:LEU	3.137	10.4	17	O	N	A:26:_:LEU			
A:22:_:LEU	VDW:SC_SC	A:44:_:TYR	4.012	-999.9	6	CD2	CG				
A:22:_:LEU	VDW:SC_SC	A:49:_:PHE	3.64	-999.9	6	CD2	CE1				
A:23:_:ARG	HBOND:MC_MC	A:26:_:LEU	3.483	54.94	17	O	N	A:26:_:LEU			
A:23:_:ARG	HBOND:MC_MC	A:27:_:THR	2.957	11.261	17	O	N	A:27:_:THR			
A:23:_:ARG	HBOND:SC_MC	A:140:_:VAL	3.444	58.519	17	NE	O	A:23:_:ARG			
A:23:_:ARG	HBOND:SC_MC	A:141:_:ASP	2.926	28.211	17	NE	O	A:23:_:ARG			
A:24:_:ASP	HBOND:MC_MC	A:27:_:THR	3.187	56.535	17	O	N	A:27:_:THR			
A:24:_:ASP	HBOND:MC_MC	A:28:_:GLU	2.881	4.92	17	O	N	A:28:_:GLU			
A:25:_:ILE	HBOND:MC_MC	A:29:_:ALA	2.856	12.603	17	O	N	A:29:_:ALA			
A:25:_:ILE	VDW:SC_SC	A:42:_:LEU	3.767	-999.9	6	CG2	CD2				
A:25:_:ILE	VDW:SC_SC	A:49:_:PHE	3.958	-999.9	6	CD1	CZ				
A:25:_:ILE	VDW:SC_SC	A:72:_:ARG	3.715	-999.9	6	CG1	CZ				
A:26:_:LEU	HBOND:MC_MC	A:30:_:ARG	2.963	14.231	17	O	N	A:30:_:ARG			
A:26:_:LEU	VDW:SC_SC	A:96:_:TYR	3.567	-999.9	6	CD1	CG				
A:26:_:LEU	VDW:SC_SC	A:140:_:VAL	3.576	-999.9	6	CD1	CG2				
A:27:_:THR	HBOND:MC_MC	A:31:_:ASP	3.305	14.03	17	O	N	A:31:_:ASP			
A:27:_:THR	VDW:SC_SC	A:143:_:VAL	3.945	-999.9	6	CG2	CG1				
A:27:_:THR	VDW:SC_MC	A:145:_:LYS	3.747	-999.9	6	CG2	C				
A:28:_:GLU	HBOND:MC_MC	A:32:_:PHE	3.08	5.92	17	O	N	A:32:_:PHE			
A:28:_:GLU	IONIC:SC_SC	A:72:_:ARG	3.482	139.606	20	-12.585,-4.665,13.627	CZ		A:72:_:ARG		
A:28:_:GLU	VDW:SC_SC	A:72:_:ARG	3.901	-999.9	6	CD	CZ				
A:29:_:ALA	HBOND:MC_MC	A:33:_:ASN	2.895	14.911	17	O	N	A:33:_:ASN			
A:29:_:ALA	VDW:MC_SC	A:33:_:ASN	3.933	-999.9	6	C	CG				
A:29:_:ALA	VDW:SC_SC	A:94:_:MET	3.939	-999.9	6	CB	CB				
A:30:_:ARG	HBOND:MC_MC	A:34:_:ASP	3.197	13.001	17	O	N	A:34:_:ASP			
A:30:_:ARG	HBOND:SC_MC	A:94:_:MET	2.805	15.983	17	NE	O	A:30:_:ARG			
A:30:_:ARG	HBOND:SC_SC	A:137:_:GLN	2.86	11.762	17	NH2	OE1	A:30:_:ARG			
A:31:_:ASP	HBOND:MC_MC	A:34:_:ASP	3.425	51.121	17	O	N	A:34:_:ASP			

A:31:_:ASP	HBOND:MC_MC	A:35:_:LEU	3.199	9.325	17	O	N	A:35:_:LEU	
A:31:_:ASP	VDW:SC_SC	A:146:_:VAL	3.44	-999.9	6	CG	CG1		
A:32:_:PHE	HBOND:MC_MC	A:35:_:LEU	3.377	54.574	17	O	N	A:35:_:LEU	
A:32:_:PHE	HBOND:MC_MC	A:36:_:ASN	3.05	39.304	17	O	N	A:36:_:ASN	
A:32:_:PHE	VDW:SC_SC	A:36:_:ASN	3.451	-999.9	6	CE2	OD1		
A:32:_:PHE	VDW:SC_SC	A:63:_:LEU	3.892	-999.9	6	CE1	CD1		
A:33:_:ASN	HBOND:MC_MC	A:36:_:ASN	3.32	28.897	17	O	N	A:36:_:ASN	
A:33:_:ASN	HBOND:MC_MC	A:37:_:GLU	3.02	55.527	17	O	N	A:37:_:GLU	
A:33:_:ASN	HBOND:MC_MC	A:38:_:ILE	2.859	2.633	17	O	N	A:38:_:ILE	
A:33:_:ASN	VDW:SC_SC	A:38:_:ILE	3.735	-999.9	6	CG	CG2		
A:33:_:ASN	VDW:SC_SC	A:63:_:LEU	3.506	-999.9	6	OD1	CD2		
A:33:_:ASN	VDW:SC_SC	A:94:_:MET	3.284	-999.9	6	ND2	CE		
A:36:_:ASN	VDW:SC_SC	A:59:_:VAL	3.973	-999.9	6	CB	CG2		
A:38:_:ILE	VDW:SC_SC	A:59:_:VAL	3.77	-999.9	6	CD1	CG2		
A:38:_:ILE	VDW:SC_SC	A:63:_:LEU	3.602	-999.9	6	CD1	CD2		
A:39:_:CYS	HBOND:MC_MC	A:54:_:GLU	2.844	11.052	17	O	N	A:54:_:GLU	
A:39:_:CYS	VDW:SC_SC	A:54:_:GLU	3.792	-999.9	6	SG	CB		
A:39:_:CYS	HBOND:SC_SC	A:87:_:ASN	3.368	47.298	17	SG	OD1	A:39:_:CYS	
A:39:_:CYS	VDW:SC_SC	A:87:_:ASN	4.004	-999.9	6	SG	CG		
A:39:_:CYS	VDW:SC_MC	A:89:_:PHE	3.962	-999.9	6	CB	C		
A:40:_:GLY	VDW:MC_SC	A:89:_:PHE	3.913	-999.9	6	C	CB		
A:40:_:GLY	HBOND:MC_MC	A:92:_:TRP	3.068	25.837	17	N	O	A:40:_:GLY	
A:40:_:GLY	VDW:MC_SC	A:94:_:MET	3.694	-999.9	6	C	SD		
A:40:_:GLY	HBOND:MC_MC	A:95:_:LYS	2.861	8.023	17	O	N	A:95:_:LYS	
A:41:_:VAL	HBOND:MC_MC	A:52:_:CYS	2.972	7.552	17	O	N	A:52:_:CYS	
A:41:_:VAL	VDW:SC_SC	A:52:_:CYS	3.891	-999.9	6	CG1	CB		
A:41:_:VAL	VDW:SC_SC	A:89:_:PHE	3.574	-999.9	6	CG1	CE1		
A:41:_:VAL	VDW:MC_SC	A:94:_:MET	3.411	-999.9	6	C	SD		
A:41:_:VAL	VDW:SC_SC	A:95:_:LYS	3.86	-999.9	6	CG2	CB		
A:41:_:VAL	VDW:SC_SC	A:97:_:VAL	3.809	-999.9	6	CG2	CG2		
A:41:_:VAL	VDW:SC_SC	A:132:_:LEU	3.793	-999.9	6	CG1	CD1		
A:42:_:LEU	VDW:SC_SC	A:49:_:PHE	3.814	-999.9	6	CD1	CD1		
A:42:_:LEU	VDW:SC_SC	A:51:_:GLN	3.775	-999.9	6	CD1	CB		
A:42:_:LEU	VDW:SC_SC	A:94:_:MET	3.902	-999.9	6	CB	SD		
A:42:_:LEU	HBOND:MC_MC	A:95:_:LYS	2.764	6.32	17	N	O	A:42:_:LEU	
A:42:_:LEU	HBOND:MC_MC	A:97:_:VAL	2.94	12.688	17	O	N	A:97:_:VAL	
A:43:_:TYR	HBOND:MC_MC	A:50:_:PHE	2.766	1.63	17	N	O	A:43:_:TYR	
A:43:_:TYR	VDW:SC_MC	A:98:_:GLN	3.875	-999.9	6	CE1	C		
A:43:_:TYR	VDW:SC_SC	A:103:_:ILE	3.903	-999.9	6	CE1	CD1		
A:43:_:TYR	VDW:SC_SC	A:117:_:PRO	3.839	-999.9	6	CE2	CG		
A:43:_:TYR	PIPISTACK:SC_SC	A:128:_:PHE	5.181	88.544	9.4	-25.316,-18.657,17.838	-24.789,-23.357,15.725		N n0.41,p5.62
A:43:_:TYR	VDW:SC_SC	A:128:_:PHE	3.73	-999.9	6	CD2	CZ		
A:43:_:TYR	VDW:SC_SC	A:132:_:LEU	3.858	-999.9	6	CB	CD2		
A:44:_:TYR	HBOND:SC_SC	A:47:_:ASN	3.171	40.799	17	OH	OD1	A:44:_:TYR	
A:44:_:TYR	VDW:SC_MC	A:47:_:ASN	3.996	-999.9	6	CE1	C		
A:44:_:TYR	VDW:SC_MC	A:48:_:ALA	3.957	-999.9	6	CD1	C		
A:44:_:TYR	PIPISTACK:SC_SC	A:49:_:PHE	5.143	125.125	9.4	-22.724,-12.207,21.598	-18.165,-12.958,19.341		T-FE n4.61,p2.07
A:44:_:TYR	VDW:SC_SC	A:49:_:PHE	3.51	-999.9	6	CD1	CE1		
A:44:_:TYR	VDW:SC_SC	A:96:_:TYR	3.883	-999.9	6	CB	CE1		
A:44:_:TYR	HBOND:MC_MC	A:97:_:VAL	3.181	16.409	17	N	O	A:44:_:TYR	
A:45:_:ALA	HBOND:MC_MC	A:48:_:ALA	2.953	47.773	17	O	N	A:48:_:ALA	
A:45:_:ALA	VDW:SC_SC	A:99:_:ARG	3.666	-999.9	6	CB	CG		
A:46:_:ASP	HBOND:SC_SC	A:99:_:ARG	2.812	55.402	17	OD1	NH1	A:99:_:ARG	
A:46:_:ASP	HBOND:SC_MC	A:115:_:PHE	2.759	13.806	17	OD2	N	A:115:_:PHE	
A:46:_:ASP	VDW:SC_SC	A:115:_:PHE	3.961	-999.9	6	CG	CB		
A:48:_:ALA	VDW:SC_SC	A:116:_:ASN	3.199	-999.9	6	CB	OD1		
A:48:_:ALA	VDW:SC_SC	A:117:_:PRO	3.779	-999.9	6	CB	CD		
A:49:_:PHE	PIPISTACK:SC_SC	A:73:_:HIS	6.195	98.829	9.4	-18.165,-12.958,19.341	-12.069,-14.019,19.022		L n4.26,p4.36
A:49:_:PHE	VDW:MC_SC	A:117:_:PRO	3.538	-999.9	6	C	CG		
A:50:_:PHE	VDW:SC_SC	A:79:_:LEU	3.725	-999.9	6	CE2	CD1		
A:50:_:PHE	VDW:SC_SC	A:117:_:PRO	3.607	-999.9	6	CB	CB		
A:50:_:PHE	VDW:SC_SC	A:125:_:LEU	3.617	-999.9	6	CE2	CD2		
A:50:_:PHE	PIPISTACK:SC_SC	A:128:_:PHE	5.205	87.607	9.4	-19.907,-22.266,14.284	-24.789,-23.357,15.725		N n0.29,p5.15
A:50:_:PHE	VDW:SC_SC	A:128:_:PHE	3.732	-999.9	6	CG	CE2		
A:50:_:PHE	VDW:SC_SC	A:129:_:LEU	3.857	-999.9	6	CE1	CD2		
A:50:_:PHE	VDW:SC_SC	A:132:_:LEU	3.632	-999.9	6	CD1	CD1		
A:51:_:GLN	VDW:SC_SC	A:67:_:ILE	3.502	-999.9	6	OE1	CD1		
A:51:_:GLN	VDW:SC_SC	A:94:_:MET	3.368	-999.9	6	NE2	CE		
A:52:_:CYS	VDW:SC_SC	A:89:_:PHE	3.851	-999.9	6	SG	CD2		
A:52:_:CYS	VDW:SC_SC	A:129:_:LEU	4.035	-999.9	6	CB	CD2		
A:53:_:LEU	VDW:SC_SC	A:60:_:VAL	4.025	-999.9	6	CB	CG1		
A:53:_:LEU	VDW:SC_SC	A:63:_:LEU	3.971	-999.9	6	CD2	CD2		
A:53:_:LEU	VDW:SC_SC	A:67:_:ILE	3.921	-999.9	6	CD1	CD1		
A:53:_:LEU	VDW:SC_SC	A:94:_:MET	3.814	-999.9	6	CD2	CE		
A:54:_:GLU	VDW:MC_SC	A:60:_:VAL	3.902	-999.9	6	C	CG2		
A:54:_:GLU	VDW:SC_SC	A:84:_:ILE	3.839	-999.9	6	CD	CD1		
A:54:_:GLU	VDW:SC_SC	A:87:_:ASN	3.761	-999.9	6	CD	OD1		
A:54:_:GLU	HBOND:SC_MC	A:88:_:SER	3.425	48.299	17	OE2	N	A:88:_:SER	
A:54:_:GLU	HBOND:SC_MC	A:89:_:PHE	2.849	20.774	17	OE1	N	A:89:_:PHE	
A:54:_:GLU	VDW:SC_SC	A:89:_:PHE	3.812	-999.9	6	CD	CB		
A:55:_:GLY	VDW:MC_SC	A:60:_:VAL	3.755	-999.9	6	C	CG2		
A:56:_:GLU	HBOND:MC_MC	A:59:_:VAL	3.25	49.899	17	O	N	A:59:_:VAL	
A:56:_:GLU	VDW:SC_SC	A:59:_:VAL	3.977	-999.9	6	CB	CG1		
A:56:_:GLU	HBOND:MC_MC	A:60:_:VAL	3.027	5.31	17	O	N	A:60:_:VAL	
A:57:_:LYS	HBOND:MC_MC	A:60:_:VAL	3.394	56.16	17	O	N	A:60:_:VAL	
A:57:_:LYS	HBOND:MC_MC	A:61:_:GLU	2.912	9.247	17	O	N	A:61:_:GLU	
A:57:_:LYS	VDW:SC_SC	A:61:_:GLU	3.567	-999.9	6	CE	CD		
A:58:_:GLU	HBOND:MC_MC	A:62:_:ARG	3.11	9.27	17	O	N	A:62:_:ARG	
A:58:_:GLU	VDW:MC_SC	A:62:_:ARG	3.703	-999.9	6	C	CZ		
A:59:_:VAL	HBOND:MC_MC	A:63:_:LEU	2.911	11.99	17	O	N	A:63:_:LEU	
A:60:_:VAL	HBOND:MC_MC	A:63:_:LEU	3.356	58.122	17	O	N	A:63:_:LEU	
A:60:_:VAL	HBOND:MC_MC	A:64:_:PHE	2.865	11.693	17	O	N	A:64:_:PHE	
A:61:_:GLU	HBOND:MC_MC	A:64:_:PHE	3.244	55.728	17	O	N	A:64:_:PHE	
A:61:_:GLU	HBOND:MC_MC	A:65:_:GLU	2.966	9.351	17	O	N	A:65:_:GLU	
A:62:_:ARG	HBOND:MC_MC	A:66:_:LYS	3.213	18.954	17	O	N	A:66:_:LYS	
A:63:_:LEU	HBOND:MC_MC	A:66:_:LYS	3.201	47.593	17	O	N	A:66:_:LYS	
A:63:_:LEU	HBOND:MC_MC	A:67:_:ILE	2.91	12.615	17	O	N	A:67:_:ILE	
A:63:_:LEU	VDW:SC_SC	A:67:_:ILE	3.751	-999.9	6	CG	CD1		
A:64:_:PHE	HBOND:MC_MC	A:67:_:ILE	3.236	56.382	17	O	N	A:67:_:ILE	
A:64:_:PHE	HBOND:MC_MC	A:68:_:GLN	2.964	13.864	17	O	N	A:68:_:GLN	
A:64:_:PHE	VDW:SC_SC	A:68:_:GLN	3.431	-999.9	6	CD2	NE2		
A:64:_:PHE	VDW:SC_SC	A:76:_:VAL	3.94	-999.9	6	CZ	CG1		
A:64:_:PHE	PIPISTACK:SC_SC	A:78:_:TRP	5.217	125.323	9.4	-8.682,-20.106,13.622	-7.751,-25.232,13.338		T-EF n2.42,p3.99

A:64:_:PHE	VDW:SC_SC	A:78:_:TRP	3.87	-999.9	6	CE2	CD2		
A:66:_:LYS	HBOND:MC_MC	A:69:_:LYS	3.258	21.904	17	O	N	A:69:_:LYS	
A:67:_:ILE	HBOND:MC_MC	A:70:_:ASP	3.014	10.94	17	O	N	A:70:_:ASP	
A:67:_:ILE	HBOND:MC_SC	A:73:_:HIS	2.729	2.12	17	O	NE2	A:73:_:HIS	
A:67:_:ILE	VDW:SC_SC	A:73:_:HIS	3.418	-999.9	6	CG2	CE1		
A:70:_:ASP	VDW:SC_SC	A:73:_:HIS	3.924	-999.9	6	CB	CD2		
A:77:_:LYS	HBOND:SC_MC	A:118:_:ILE	2.817	37.578	17	NZ	O	A:77:_:LYS	
A:77:_:LYS	HBOND:SC_MC	A:120:_:LEU	2.993	60.333	17	NZ	O	A:77:_:LYS	
A:78:_:TRP	HBOND:SC_SC	A:81:_:THR	2.975	30.765	17	NE1	OG1	A:78:_:TRP	
A:79:_:LEU	VDW:MC_SC	A:122:_:GLN	3.852	-999.9	6	C	NE2		
A:80:_:CYS	VDW:SC_SC	A:125:_:LEU	3.579	-999.9	6	SG	CD1		
A:87:_:ASN	HBOND:MC_MC	A:90:_:GLN	3.259	13.377	17	O	N	A:90:_:GLN	
A:87:_:ASN	VDW:SC_SC	A:90:_:GLN	3.305	-999.9	6	ND2	CB		
A:89:_:PHE	HBOND:MC_MC	A:92:_:TRP	2.887	17.865	17	O	N	A:92:_:TRP	
A:89:_:PHE	VDW:SC_SC	A:92:_:TRP	3.861	-999.9	6	CD1	CZ3		
A:92:_:TRP	VDW:SC_SC	A:95:_:LYS	3.554	-999.9	6	CG	CB		
A:92:_:TRP	VDW:SC_SC	A:133:_:LEU	3.797	-999.9	6	CZ3	CD1		
A:92:_:TRP	VDW:SC_SC	A:137:_:GLN	3.747	-999.9	6	CD1	NE2		
A:95:_:LYS	HBOND:SC_MC	A:132:_:LEU	2.715	30.914	17	NZ	O	A:95:_:LYS	
A:95:_:LYS	HBOND:SC_MC	A:133:_:LEU	2.823	58.397	17	NZ	O	A:95:_:LYS	
A:95:_:LYS	HBOND:SC_MC	A:135:_:ALA	2.722	22.189	17	NZ	O	A:95:_:LYS	
A:95:_:LYS	VDW:MC_SC	A:137:_:GLN	3.645	-999.9	6	C	OE1		
A:96:_:TYR	HBOND:MC_SC	A:137:_:GLN	3.031	25.834	17	N	OE1	A:96:_:TYR	
A:96:_:TYR	HBOND:MC_MC	A:138:_:THR	2.881	18.811	17	O	N	A:138:_:THR	
A:96:_:TYR	VDW:SC_SC	A:138:_:THR	3.723	-999.9	6	CD2	CB		
A:96:_:TYR	VDW:SC_SC	A:140:_:VAL	3.461	-999.9	6	CD2	CG1		
A:97:_:VAL	VDW:SC_SC	A:103:_:ILE	4.035	-999.9	6	CG1	CD1		
A:97:_:VAL	VDW:SC_SC	A:132:_:LEU	3.876	-999.9	6	CG1	CD2		
A:97:_:VAL	VDW:SC_SC	A:135:_:ALA	3.705	-999.9	6	CG1	CB		
A:98:_:GLN	HBOND:MC_SC	A:138:_:THR	3.291	42.871	17	N	OG1	A:98:_:GLN	
A:98:_:GLN	VDW:SC_SC	A:138:_:THR	3.929	-999.9	6	NE2	CG2		
A:99:_:ARG	HBOND:SC_SC	A:104:_:GLU	2.647	20.199	17	NE	OE2	A:99:_:ARG	
A:99:_:ARG	VDW:SC_SC	A:115:_:PHE	3.512	-999.9	6	CZ	CD2		
A:100:_:ASN	HBOND:SC_MC	A:103:_:ILE	2.845	5.918	17	OD1	N	A:103:_:ILE	
A:100:_:ASN	VDW:SC_SC	A:103:_:ILE	3.351	-999.9	6	OD1	CG1		
A:100:_:ASN	HBOND:MC_MC	A:104:_:GLU	2.956	6.606	17	O	N	A:104:_:GLU	
A:100:_:ASN	VDW:SC_SC	A:135:_:ALA	3.6	-999.9	6	ND2	CB		
A:101:_:THR	HBOND:MC_MC	A:105:_:ALA	2.87	11.697	17	O	N	A:105:_:ALA	
A:102:_:ASN	HBOND:MC_MC	A:105:_:ALA	3.375	58.621	17	O	N	A:105:_:ALA	
A:102:_:ASN	HBOND:MC_MC	A:106:_:PHE	2.912	14.336	17	O	N	A:106:_:PHE	
A:102:_:ASN	VDW:SC_SC	A:131:_:GLU	3.851	-999.9	6	ND2	CD		
A:103:_:ILE	HBOND:MC_MC	A:106:_:PHE	3.143	54.429	17	O	N	A:106:_:PHE	
A:103:_:ILE	HBOND:MC_MC	A:107:_:PHE	3.067	7.691	17	O	N	A:107:_:PHE	
A:103:_:ILE	VDW:SC_SC	A:107:_:PHE	3.804	-999.9	6	CG2	CE2		
A:103:_:ILE	VDW:SC_SC	A:128:_:PHE	3.978	-999.9	6	CG2	CE1		
A:104:_:GLU	HBOND:MC_MC	A:107:_:PHE	3.462	55.668	17	O	N	A:107:_:PHE	
A:104:_:GLU	HBOND:MC_MC	A:108:_:LEU	3.014	7.289	17	O	N	A:108:_:LEU	
A:104:_:GLU	VDW:SC_SC	A:115:_:PHE	3.796	-999.9	6	CG	CE1		
A:105:_:ALA	HBOND:MC_MC	A:108:_:LEU	3.489	59.861	17	O	N	A:108:_:LEU	
A:105:_:ALA	HBOND:MC_MC	A:109:_:LYS	2.905	13.506	17	O	N	A:109:_:LYS	
A:106:_:PHE	HBOND:MC_MC	A:110:_:MET	3.424	32.014	17	O	N	A:110:_:MET	
A:106:_:PHE	VDW:SC_SC	A:110:_:MET	3.842	-999.9	6	CE2	CE		
A:106:_:PHE	VDW:SC_SC	A:120:_:LEU	3.903	-999.9	6	CE2	CD2		
A:106:_:PHE	VDW:SC_SC	A:124:_:ASN	4.012	-999.9	6	CZ	CB		
A:106:_:PHE	PIPISTACK:SC_SC	A:127:_:PHE	6.155	40.038	9.4	-28.348,-28.413,17.160	-29.274,-32.537,12.686		T-EF n2.46,p6.41
A:106:_:PHE	VDW:SC_SC	A:127:_:PHE	3.447	-999.9	6	CE1	CB		
A:106:_:PHE	PIPISTACK:SC_SC	A:128:_:PHE	6.348	153.763	9.4	-28.348,-28.413,17.160	-24.789,-23.357,15.725		P n3.47,p5.64
A:107:_:PHE	HBOND:MC_MC	A:110:_:MET	3.386	30.054	17	O	N	A:110:_:MET	
A:107:_:PHE	HBOND:MC_MC	A:111:_:GLY	3.298	49.886	17	O	N	A:111:_:GLY	
A:107:_:PHE	HBOND:MC_MC	A:112:_:GLU	2.976	22.874	17	O	N	A:112:_:GLU	
A:107:_:PHE	VDW:SC_SC	A:112:_:GLU	3.916	-999.9	6	CD1	CB		
A:107:_:PHE	PIPISTACK:SC_SC	A:115:_:PHE	5.933	79.073	9.4	-26.614,-24.520,21.332	-30.361,-21.464,24.771		N n1.78,p6.61
A:107:_:PHE	VDW:SC_SC	A:115:_:PHE	3.619	-999.9	6	CB	CD1		
A:107:_:PHE	VDW:SC_SC	A:120:_:LEU	3.917	-999.9	6	CZ	CD2		
A:107:_:PHE	PIPISTACK:SC_SC	A:128:_:PHE	6.01	93.808	9.4	-26.614,-24.520,21.332	-24.789,-23.357,15.725		N n2.25,p5.67
A:107:_:PHE	VDW:SC_SC	A:128:_:PHE	3.873	-999.9	6	CE2	CE1		
A:108:_:LEU	HBOND:MC_MC	A:111:_:GLY	3.121	21.702	17	O	N	A:111:_:GLY	
A:110:_:MET	VDW:SC_SC	A:120:_:LEU	3.839	-999.9	6	CE	CD2		
A:110:_:MET	VDW:SC_SC	A:124:_:ASN	3.969	-999.9	6	SD	CG		
A:112:_:GLU	VDW:SC_SC	A:119:_:LEU	3.975	-999.9	6	CD	CD1		
A:116:_:ASN	HBOND:MC_MC	A:119:_:LEU	3.239	14.688	17	O	N	A:119:_:LEU	
A:117:_:PRO	HBOND:MC_MC	A:120:_:LEU	3.119	17.459	17	O	N	A:120:_:LEU	
A:117:_:PRO	VDW:SC_SC	A:120:_:LEU	3.978	-999.9	6	CB	CD1		
A:120:_:LEU	VDW:MC_SC	A:124:_:ASN	3.74	-999.9	6	C	OD1		
A:120:_:LEU	VDW:SC_SC	A:128:_:PHE	3.361	-999.9	6	CD1	CD2		
A:121:_:ASN	HBOND:MC_SC	A:124:_:ASN	2.854	3.348	17	N	OD1	A:121:_:ASN	
A:121:_:ASN	VDW:SC_SC	A:124:_:ASN	3.213	-999.9	6	CG	OD1		
A:122:_:GLN	HBOND:MC_MC	A:125:_:LEU	3.235	23.171	17	O	N	A:125:_:LEU	
A:123:_:GLN	HBOND:MC_MC	A:126:_:LYS	3.481	24.664	17	O	N	A:126:_:LYS	
A:124:_:ASN	HBOND:MC_MC	A:127:_:PHE	3.236	48.866	17	O	N	A:127:_:PHE	
A:124:_:ASN	HBOND:MC_MC	A:128:_:PHE	2.873	5.949	17	O	N	A:128:_:PHE	
A:125:_:LEU	HBOND:MC_MC	A:129:_:LEU	2.935	13.442	17	O	N	A:129:_:LEU	
A:127:_:PHE	HBOND:MC_MC	A:130:_:ASN	3.206	54.944	17	O	N	A:130:_:ASN	
A:127:_:PHE	HBOND:MC_MC	A:131:_:GLU	2.918	10.451	17	O	N	A:131:_:GLU	
A:128:_:PHE	HBOND:MC_MC	A:131:_:GLU	3.214	56.348	17	O	N	A:131:_:GLU	
A:128:_:PHE	HBOND:MC_MC	A:132:_:LEU	2.963	8.845	17	O	N	A:132:_:LEU	
A:128:_:PHE	VDW:SC_SC	A:132:_:LEU	3.742	-999.9	6	CE2	CD1		
A:129:_:LEU	HBOND:MC_MC	A:132:_:LEU	3.43	58.336	17	O	N	A:132:_:LEU	
A:129:_:LEU	HBOND:MC_MC	A:133:_:LEU	2.902	17.007	17	O	N	A:133:_:LEU	
A:130:_:ASN	HBOND:MC_MC	A:133:_:LEU	3.159	52.567	17	O	N	A:133:_:LEU	
A:130:_:ASN	HBOND:MC_MC	A:134:_:ILE	2.928	14.801	17	O	N	A:134:_:ILE	
A:131:_:GLU	HBOND:MC_MC	A:134:_:ILE	3.459	49.567	17	O	N	A:134:_:ILE	
A:131:_:GLU	HBOND:MC_MC	A:135:_:ALA	2.917	25.177	17	O	N	A:135:_:ALA	
A:143:_:VAL	VDW:SC_SC	A:148:_:MET	3.476	-999.9	6	CG2	CE		
A:143:_:VAL	VDW:SC_SC	A:149:_:VAL	3.683	-999.9	6	CG1	CG1		
A:145:_:LYS	HBOND:MC_MC	A:148:_:MET	3.354	16.252	17	O	N	A:148:_:MET	
A:145:_:LYS	VDW:SC_SC	A:148:_:MET	3.848	-999.9	6	CG	CG		
A:146:_:VAL	HBOND:MC_MC	A:149:_:VAL	3.18	4.967	17	O	N	A:149:_:VAL	
A:147:_:GLY	HBOND:MC_SC	A:150:_:ASN	3.267	36.012	17	O	ND2	A:150:_:ASN	
A:147:_:GLY	VDW:MC_SC	A:150:_:ASN	3.748	-999.9	6	C	ND2		
**LIGAND INTERACTIONS**									
_:1:_:FMN	IAC:LIG_SC	A:7:_:TYR	2.978	-999.9	0	C7M	HH		
_:1:_:FMN	IAC:LIG_SC	A:9:_:SER	4.83	-999.9	0	C7M	OG		

_:1:_:FMN	IAC:LIG_SC	A:11:_:ARG	6.582	-999.9	0	C8M	HA
_:1:_:FMN	IAC:LIG_SC	A:12:_:ASN	6.808	-999.9	0	C8M	H
_:1:_:FMN	IAC:LIG_SC	A:21:_:ASP	6.546	-999.9	0	C8M	HB3
_:1:_:FMN	IAC:LIG_SC	A:22:_:LEU	6.578	-999.9	0	C7M	HD22
_:1:_:FMN	IAC:LIG_MC	A:24:_:ASP	6.63	-999.9	0	C1'	O
_:1:_:FMN	VDW:LIG_SC	A:25:_:ILE	3.762	-999.9	6	C6	CG2
_:1:_:FMN	IAC:LIG_SC	A:26:_:LEU	6.272	-999.9	0	N5	HD22
_:1:_:FMN	IAC:LIG_SC	A:28:_:GLU	2.618	-999.9	0	O3'	OE1
_:1:_:FMN	VDW:LIG_SC	A:29:_:ALA	3.363	-999.9	6	C4	CB
_:1:_:FMN	IAC:LIG_MC	A:30:_:ARG	5.475	-999.9	0	N3	N
_:1:_:FMN	IAC:LIG_MC	A:31:_:ASP	6.144	-999.9	0	O2	C
_:1:_:FMN	IAC:LIG_SC	A:32:_:PHE	2.246	-999.9	0	O2	HB3
_:1:_:FMN	VDW:LIG_SC	A:33:_:ASN	3.648	-999.9	6	C4	OD1
_:1:_:FMN	IAC:LIG_SC	A:36:_:ASN	6.639	-999.9	0	O2	HB2
_:1:_:FMN	IAC:LIG_SC	A:38:_:ILE	4.531	-999.9	0	O4	HG21
_:1:_:FMN	IAC:LIG_MC	A:39:_:CYS	6.676	-999.9	0	O4	O
_:1:_:FMN	IAC:LIG_SC	A:40:_:GLY	6.407	-999.9	0	O4	HA1
_:1:_:FMN	IAC:LIG_SC	A:42:_:LEU	3.188	-999.9	0	C7M	HD11
_:1:_:FMN	IAC:LIG_MC	A:43:_:TYR	6.912	-999.9	0	C7M	O
_:1:_:FMN	IAC:LIG_SC	A:44:_:TYR	6.674	-999.9	0	C7M	HD1
_:1:_:FMN	IAC:LIG_SC	A:49:_:PHE	3.856	-999.9	0	C7M	CG
_:1:_:FMN	IAC:LIG_MC	A:50:_:PHE	6.614	-999.9	0	C7M	O
_:1:_:FMN	VDW:LIG_SC	A:51:_:GLN	3.333	-999.9	6	C6	OE1
_:1:_:FMN	IAC:LIG_MC	A:52:_:CYS	6.301	-999.9	0	O4	O
_:1:_:FMN	IAC:LIG_SC	A:53:_:LEU	2.931	-999.9	0	O4	HD23
_:1:_:FMN	VDW:LIG_SC	A:63:_:LEU	3.54	-999.9	6	C2	CD1
_:1:_:FMN	IAC:LIG_SC	A:66:_:LYS	2.117	-999.9	0	O4'	HZ3
_:1:_:FMN	IAC:LIG_SC	A:67:_:ILE	2.659	-999.9	0	O2'	HA
_:1:_:FMN	IAC:LIG_MC	A:68:_:GLN	5.772	-999.9	0	O2'	N
_:1:_:FMN	IAC:LIG_SC	A:69:_:LYS	5.653	-999.9	0	O2'	H
_:1:_:FMN	IAC:LIG_SC	A:70:_:ASP	2.604	-999.9	0	O2'	HB2
_:1:_:FMN	IAC:LIG_SC	A:71:_:GLN	6.133	-999.9	0	O2'	H
_:1:_:FMN	IAC:LIG_SC	A:72:_:ARG	2.427	-999.9	0	O3'	HH22
_:1:_:FMN	IAC:LIG_SC	A:73:_:HIS	3.75	-999.9	0	C7M	ND1
_:1:_:FMN	IAC:LIG_MC	A:93:_:SER	5.426	-999.9	0	O4	O
_:1:_:FMN	IAC:LIG_SC	A:94:_:MET	2.678	-999.9	0	O4	HE1
_:1:_:FMN	IAC:LIG_SC	A:146:_:VAL	6.228	-999.9	0	C5'	HG21

NodeId1	Interaction	NodeId2	Distance(*)	Angle	Energy	Atom1	Atom2	Donor	Positive	Cation	Orientation
**AnBLUF46 LIGHT**											
A:2:_:SER	HBOND:MC_MC	A:58:_:LYS	3.166	22.1	17	O	N	A:58:_:LYS			
A:3:_:LEU	VDW:SC_MC	A:56:_:GLY	3.747	-999.9	6	CD2	C				
A:3:_:LEU	VDW:SC_SC	A:57:_:GLU	4.019	-999.9	6	CD2	CB				
A:3:_:LEU	HBOND:MC_MC	A:86:_:SER	3.002	13.078	17	O	N	A:86:_:SER			
A:3:_:LEU	VDW:SC_SC	A:89:_:ARG	4.018	-999.9	6	CD1	CZ				
A:4:_:ILE	HBOND:MC_MC	A:56:_:GLY	2.826	6.214	17	N	O	A:4:_:ILE			
A:4:_:ILE	VDW:SC_SC	A:58:_:LYS	4.003	-999.9	6	CD1	CG				
A:4:_:ILE	VDW:SC_SC	A:61:_:VAL	3.796	-999.9	6	CD1	CG1				
A:4:_:ILE	VDW:SC_SC	A:83:_:LEU	3.582	-999.9	6	CD1	CD1				
A:5:_:GLY	VDW:MC_SC	A:53:_:TYR	3.79	-999.9	6	C	CE2				
A:5:_:GLY	HBOND:MC_MC	A:84:_:GLU	2.937	14.412	17	N	O	A:5:_:GLY			
A:6:_:PHE	VDW:MC_SC	A:53:_:TYR	3.751	-999.9	6	C	CD2				
A:6:_:PHE	HBOND:MC_MC	A:54:_:LEU	2.813	8.791	17	N	O	A:6:_:PHE			
A:6:_:PHE	VDW:SC_SC	A:61:_:VAL	3.637	-999.9	6	CB	CG1				
A:6:_:PHE	PIPISTACK:SC_SC	A:65:_:PHE	4.48	140.848	9.4	-3.609,-5.368,29.146	-2.070,-2.261,26.309				T-EF n2.76,p3.59
A:6:_:PHE	VDW:SC_SC	A:65:_:PHE	3.194	-999.9	6	CE1	CD2				
A:6:_:PHE	VDW:SC_SC	A:80:_:ILE	3.479	-999.9	6	CZ	CG2				
A:6:_:PHE	VDW:SC_SC	A:83:_:LEU	3.596	-999.9	6	CD2	CD1				
A:7:_:MET	VDW:SC_SC	A:51:_:LEU	3.694	-999.9	6	CE	CD2				
A:7:_:MET	VDW:SC_SC	A:53:_:TYR	3.822	-999.9	6	CE	CD2				
A:7:_:MET	HBOND:MC_MC	A:81:_:ILE	2.841	11.496	17	O	N	A:81:_:ILE			
A:7:_:MET	VDW:SC_SC	A:81:_:ILE	3.906	-999.9	6	CG	CD1				
A:7:_:MET	HBOND:MC_MC	A:82:_:PHE	2.887	6.519	17	N	O	A:7:_:MET			
A:7:_:MET	VDW:SC_SC	A:127:_:ILE	3.347	-999.9	6	SD	CG2				
A:7:_:MET	VDW:SC_SC	A:131:_:ILE	3.557	-999.9	6	CE	CG1				
A:8:_:TYR	VDW:MC_SC	A:51:_:LEU	3.833	-999.9	6	C	CD1				
A:8:_:TYR	HBOND:MC_MC	A:52:_:GLN	2.987	9.232	17	O	N	A:52:_:GLN			
A:8:_:TYR	VDW:SC_SC	A:52:_:GLN	3.263	-999.9	6	CE2	OE1				
A:8:_:TYR	PIPISTACK:SC_SC	A:65:_:PHE	5.809	56.525	9.4	0.812,-0.382,30.989	-2.070,-2.261,26.309				L n4.51,p3.50
A:8:_:TYR	VDW:SC_SC	A:65:_:PHE	3.869	-999.9	6	CD2	CD2				
A:8:_:TYR	VDW:SC_SC	A:69:_:ILE	3.552	-999.9	6	CZ	CG2				
A:8:_:TYR	PIPISTACK:SC_SC	A:75:_:HIS	6	103.584	9.4	0.812,-0.382,30.989	3.513,4.507,28.797				N n2.80,p6.41
A:8:_:TYR	VDW:SC_SC	A:75:_:HIS	3.847	-999.9	6	CE1	CE1				
A:8:_:TYR	VDW:SC_SC	A:78:_:CYS	3.304	-999.9	6	CD1	SG				
A:8:_:TYR	VDW:SC_MC	A:79:_:GLU	4.031	-999.9	6	CB	C				
A:8:_:TYR	VDW:MC_SC	A:81:_:ILE	3.902	-999.9	6	C	CD1				
A:9:_:ALA	VDW:SC_SC	A:51:_:LEU	3.799	-999.9	6	CB	CD1				
A:9:_:ALA	HBOND:MC_MC	A:79:_:GLU	2.807	5.127	17	O	N	A:79:_:GLU			
A:9:_:ALA	VDW:SC_SC	A:81:_:ILE	3.831	-999.9	6	CB	CD1				
A:10:_:SER	VDW:MC_SC	A:49:_:TYR	3.797	-999.9	6	C	CD1				
A:10:_:SER	HBOND:MC_MC	A:50:_:PHE	2.834	18.929	17	O	N	A:50:_:PHE			
A:11:_:LYS	VDW:SC_SC	A:49:_:TYR	3.542	-999.9	6	CB	CE1				
A:11:_:LYS	HBOND:MC_MC	A:76:_:GLN	2.797	12.373	17	N	O	A:11:_:LYS			
A:12:_:THR	HBOND:MC_MC	A:48:_:GLY	2.829	18.228	17	N	O	A:12:_:THR			
A:12:_:THR	VDW:SC_MC	A:48:_:GLY	3.885	-999.9	6	CB	C				
A:13:_:ASN	HBOND:MC_MC	A:16:_:HIS	3.312	26.707	17	O	N	A:16:_:HIS			
A:13:_:ASN	VDW:SC_SC	A:16:_:HIS	4.031	-999.9	6	CB	CD2				
A:13:_:ASN	HBOND:MC_MC	A:74:_:ARG	3.225	8.799	17	N	O	A:13:_:ASN			
A:13:_:ASN	HBOND:SC_SC	A:76:_:GLN	3.393	60.399	17	ND2	OE1	A:13:_:ASN			
A:13:_:ASN	VDW:SC_SC	A:76:_:GLN	3.216	-999.9	6	CG	OE1				
A:16:_:HIS	HBOND:SC_MC	A:73:_:SER	2.816	28.23	17	NE2	O	A:16:_:HIS			
A:18:_:GLN	HBOND:MC_MC	A:21:_:GLN	2.965	42.99	17	O	N	A:21:_:GLN			
A:18:_:GLN	HBOND:MC_MC	A:22:_:ASP	3.022	6.997	17	O	N	A:22:_:ASP			
A:19:_:ILE	HBOND:MC_MC	A:22:_:ASP	3.321	62.008	17	O	N	A:22:_:ASP			
A:19:_:ILE	HBOND:MC_MC	A:23:_:LEU	2.881	6.971	17	O	N	A:23:_:LEU			
A:19:_:ILE	VDW:SC_SC	A:23:_:LEU	3.828	-999.9	6	CG2	CD1				
A:20:_:LYS	HBOND:MC_MC	A:24:_:ILE	3.205	12.409	17	O	N	A:24:_:ILE			
A:21:_:GLN	HBOND:MC_MC	A:24:_:ILE	3.123	55.091	17	O	N	A:24:_:ILE			
A:21:_:GLN	HBOND:MC_MC	A:25:_:ASP	2.846	8.399	17	O	N	A:25:_:ASP			
A:21:_:GLN	VDW:SC_SC	A:25:_:ASP	3.488	-999.9	6	NE2	CG				
A:22:_:ASP	HBOND:MC_MC	A:25:_:ASP	3.37	56.952	17	O	N	A:25:_:ASP			
A:22:_:ASP	HBOND:MC_MC	A:26:_:ILE	2.839	5.604	17	O	N	A:26:_:ILE			
A:22:_:ASP	VDW:MC_SC	A:26:_:ILE	3.939	-999.9	6	C	CD1				
A:22:_:ASP	VDW:SC_SC	A:74:_:ARG	3.947	-999.9	6	CG	CD				
A:23:_:LEU	HBOND:MC_MC	A:27:_:LEU	3.044	9.577	17	O	N	A:27:_:LEU			
A:23:_:LEU	VDW:SC_SC	A:50:_:PHE	3.632	-999.9	6	CD2	CE1				
A:23:_:LEU	VDW:SC_SC	A:98:_:PHE	3.941	-999.9	6	CD2	CZ				
A:24:_:ILE	HBOND:MC_MC	A:27:_:LEU	3.424	56.408	17	O	N	A:27:_:LEU			
A:24:_:ILE	HBOND:MC_MC	A:28:_:THR	2.89	10.103	17	O	N	A:28:_:THR			
A:25:_:ASP	HBOND:MC_MC	A:28:_:THR	3.235	57.583	17	O	N	A:28:_:THR			
A:25:_:ASP	HBOND:MC_MC	A:29:_:GLU	2.882	4.578	17	O	N	A:29:_:GLU			
A:25:_:ASP	VDW:MC_SC	A:29:_:GLU	3.884	-999.9	6	C	CD				
A:26:_:ILE	HBOND:MC_MC	A:30:_:ALA	2.893	13.074	17	O	N	A:30:_:ALA			
A:26:_:ILE	VDW:SC_SC	A:43:_:LEU	3.895	-999.9	6	CG2	CD2				
A:26:_:ILE	VDW:SC_SC	A:50:_:PHE	3.988	-999.9	6	CD1	CZ				
A:26:_:ILE	VDW:SC_SC	A:74:_:ARG	3.67	-999.9	6	CG1	CZ				
A:27:_:LEU	HBOND:MC_MC	A:30:_:ALA	3.452	59.787	17	O	N	A:30:_:ALA			
A:27:_:LEU	HBOND:MC_MC	A:31:_:VAL	2.835	8.355	17	O	N	A:31:_:VAL			
A:27:_:LEU	VDW:SC_SC	A:98:_:PHE	3.499	-999.9	6	CD1	CG				
A:28:_:THR	HBOND:MC_MC	A:32:_:LYS	3.178	11.82	17	O	N	A:32:_:LYS			
A:29:_:GLU	HBOND:MC_MC	A:33:_:PHE	2.997	8.322	17	O	N	A:33:_:PHE			
A:29:_:GLU	IONIC:SC_SC	A:74:_:ARG	3.465	138.227	20	-2.275,13.346,31.332	CZ		A:74:_:ARG		
A:29:_:GLU	VDW:SC_SC	A:74:_:ARG	3.888	-999.9	6	CD	CZ				
A:30:_:ALA	HBOND:MC_MC	A:33:_:PHE	3.407	57.911	17	O	N	A:33:_:PHE			
A:30:_:ALA	HBOND:MC_MC	A:34:_:ASN	2.859	11.357	17	O	N	A:34:_:ASN			
A:30:_:ALA	VDW:MC_SC	A:34:_:ASN	4.004	-999.9	6	C	CG				
A:31:_:VAL	HBOND:MC_MC	A:35:_:SER	3.186	15.85	17	O	N	A:35:_:SER			
A:31:_:VAL	VDW:SC_SC	A:96:_:MET	4	-999.9	6	CG2	CB				
A:32:_:LYS	HBOND:SC_SC	A:36:_:GLN	2.804	43.322	17	NZ	OE1	A:32:_:LYS			
A:32:_:LYS	VDW:SC_SC	A:36:_:GLN	3.445	-999.9	6	CG	OE1				
A:33:_:PHE	HBOND:MC_MC	A:36:_:GLN	3.139	47.433	17	O	N	A:36:_:GLN			
A:33:_:PHE	HBOND:MC_MC	A:37:_:ASN	3.052	48.595	17	O	N	A:37:_:ASN			
A:33:_:PHE	VDW:SC_SC	A:37:_:ASN	3.438	-999.9	6	CE1	ND2				
A:33:_:PHE	VDW:SC_SC	A:60:_:GLN	3.464	-999.9	6	CZ	NE2				
A:34:_:ASN	HBOND:MC_MC	A:37:_:ASN	3.277	21.416	17	O	N	A:37:_:ASN			
A:34:_:ASN	HBOND:MC_MC	A:38:_:ASP	3.17	59.165	17	O	N	A:38:_:ASP			
A:34:_:ASN	HBOND:MC_MC	A:39:_:ILE	2.995	5.823	17	O	N	A:39:_:ILE			
A:34:_:ASN	VDW:SC_SC	A:39:_:ILE	3.705	-999.9	6	CG	CG2				
A:34:_:ASN	VDW:SC_SC	A:64:_:LEU	3.369	-999.9	6	OD1	CD2				

A:34:_:ASN	VDW:SC_MC	A:95:_:SER	4.023	-999.9	6	CB	C			
A:34:_:ASN	VDW:SC_SC	A:96:_:MET	3.357	-999.9	6	ND2	CE			
A:38:_:ASP	IONIC:SC_SC	A:89:_:ARG	3.414	102.669	20	-17.238,0.165,36.628	CZ		A:89:_:ARG	
A:38:_:ASP	VDW:SC_SC	A:89:_:ARG	3.803	-999.9	6	CG	CZ			
A:39:_:ILE	VDW:SC_SC	A:60:_:GLN	3.9	-999.9	6	CD1	CG			
A:39:_:ILE	VDW:SC_SC	A:64:_:LEU	3.539	-999.9	6	CD1	CD2			
A:40:_:THR	HBOND:MC_MC	A:55:_:GLU	2.777	5.348	17	O	N	A:55:_:GLU		
A:40:_:THR	VDW:SC_MC	A:91:_:PHE	3.896	-999.9	6	CB	C			
A:41:_:GLY	VDW:MC_SC	A:91:_:PHE	3.84	-999.9	6	C	CB			
A:41:_:GLY	HBOND:MC_MC	A:94:_:TRP	3.104	28.146	17	N	O	A:41:_:GLY		
A:41:_:GLY	VDW:MC_SC	A:96:_:MET	3.803	-999.9	6	C	SD			
A:41:_:GLY	HBOND:MC_MC	A:97:_:LYS	2.838	6.726	17	O	N	A:97:_:LYS		
A:42:_:VAL	HBOND:MC_MC	A:53:_:TYR	2.872	9.639	17	O	N	A:53:_:TYR		
A:42:_:VAL	VDW:SC_SC	A:53:_:TYR	3.888	-999.9	6	CG1	CB			
A:42:_:VAL	VDW:SC_SC	A:91:_:PHE	3.404	-999.9	6	CG1	CZ			
A:42:_:VAL	VDW:MC_SC	A:96:_:MET	3.408	-999.9	6	C	SD			
A:42:_:VAL	VDW:SC_SC	A:97:_:LYS	3.826	-999.9	6	CG2	CB			
A:42:_:VAL	VDW:SC_SC	A:134:_:LEU	3.904	-999.9	6	CG1	CD1			
A:43:_:LEU	VDW:SC_SC	A:50:_:PHE	3.814	-999.9	6	CD1	CD1			
A:43:_:LEU	VDW:SC_SC	A:52:_:GLN	3.84	-999.9	6	CD1	CB			
A:43:_:LEU	VDW:SC_SC	A:96:_:MET	3.798	-999.9	6	CB	SD			
A:43:_:LEU	HBOND:MC_MC	A:97:_:LYS	2.764	5.909	17	N	O	A:43:_:LEU		
A:43:_:LEU	HBOND:MC_MC	A:99:_:ALA	2.979	27.883	17	O	N	A:99:_:ALA		
A:44:_:TYR	HBOND:MC_MC	A:51:_:LEU	2.768	5.741	17	N	O	A:44:_:TYR		
A:44:_:TYR	VDW:SC_MC	A:99:_:ALA	4.033	-999.9	6	CD1	C			
A:44:_:TYR	VDW:SC_MC	A:100:_:PRO	3.522	-999.9	6	CE1	C			
A:44:_:TYR	VDW:SC_SC	A:105:_:ILE	3.807	-999.9	6	CE1	CD1			
A:44:_:TYR	PIPISTACK:SC_SC	A:130:_:PHE	5.254	93.287	9.4	6.758,-0.530,41.872	5.428,-5.601,42.203			N n5.20,p0.15
A:44:_:TYR	VDW:SC_SC	A:130:_:PHE	3.967	-999.9	6	CD2	CZ			
A:44:_:TYR	VDW:SC_SC	A:134:_:LEU	3.614	-999.9	6	CD1	CD2			
A:45:_:TYR	HBOND:SC_MC	A:48:_:GLY	3.409	40.905	17	OH	N	A:48:_:GLY		
A:45:_:TYR	VDW:SC_MC	A:49:_:TYR	3.963	-999.9	6	CD1	C			
A:45:_:TYR	PIPISTACK:SC_SC	A:50:_:PHE	5.075	127.156	9.4	8.957,6.181,38.219	5.686,5.556,34.389			T-FE n4.66,p1.93
A:45:_:TYR	VDW:SC_SC	A:50:_:PHE	3.634	-999.9	6	CD1	CE1			
A:45:_:TYR	VDW:SC_SC	A:98:_:PHE	3.845	-999.9	6	CB	CE1			
A:45:_:TYR	HBOND:MC_MC	A:99:_:ALA	2.966	12.926	17	N	O	A:45:_:TYR		
A:46:_:GLY	HBOND:MC_MC	A:49:_:TYR	3.073	19.233	17	O	N	A:49:_:TYR		
A:50:_:PHE	PIPISTACK:SC_SC	A:75:_:HIS	6.091	96.301	9.4	5.686,5.556,34.389	3.513,4.507,28.797			L n4.54,p4.03
A:50:_:PHE	VDW:SC_SC	A:75:_:HIS	3.988	-999.9	6	CD2	CB			
A:51:_:LEU	VDW:SC_SC	A:81:_:ILE	3.397	-999.9	6	CD1	CD1			
A:51:_:LEU	VDW:SC_SC	A:119:_:PRO	3.675	-999.9	6	CD1	CB			
A:51:_:LEU	VDW:SC_SC	A:130:_:PHE	3.368	-999.9	6	CD2	CE2			
A:52:_:GLN	VDW:SC_SC	A:69:_:ILE	3.344	-999.9	6	OE1	CD1			
A:52:_:GLN	VDW:SC_SC	A:96:_:MET	3.403	-999.9	6	NE2	CE			
A:53:_:TYR	PIPISTACK:SC_SC	A:91:_:PHE	6.047	121.086	9.4	-4.339,-6.173,37.784	-4.651,-3.117,42.992			T-EF n2.75,p5.05
A:53:_:TYR	VDW:SC_SC	A:91:_:PHE	3.838	-999.9	6	CD1	CE2			
A:53:_:TYR	VDW:SC_SC	A:131:_:ILE	3.889	-999.9	6	CE2	CD1			
A:54:_:LEU	VDW:SC_SC	A:61:_:VAL	3.873	-999.9	6	CB	CG1			
A:54:_:LEU	VDW:SC_SC	A:64:_:LEU	3.798	-999.9	6	CD2	CD2			
A:54:_:LEU	VDW:SC_SC	A:69:_:ILE	3.748	-999.9	6	CD1	CD1			
A:54:_:LEU	VDW:SC_SC	A:96:_:MET	3.718	-999.9	6	CD2	CE			
A:55:_:GLU	VDW:MC_SC	A:61:_:VAL	3.967	-999.9	6	C	CG2			
A:55:_:GLU	VDW:SC_MC	A:89:_:ARG	3.87	-999.9	6	CD	C			
A:55:_:GLU	HBOND:SC_MC	A:90:_:LEU	2.759	23.375	17	OE2	N	A:90:_:LEU		
A:55:_:GLU	HBOND:SC_MC	A:91:_:PHE	2.792	12.835	17	OE1	N	A:91:_:PHE		
A:55:_:GLU	VDW:SC_SC	A:91:_:PHE	3.919	-999.9	6	CD	CD2			
A:56:_:GLY	VDW:MC_SC	A:61:_:VAL	3.677	-999.9	6	C	CG2			
A:57:_:GLU	HBOND:SC_MC	A:60:_:GLN	3.045	9.413	17	OE1	N	A:60:_:GLN		
A:57:_:GLU	HBOND:MC_MC	A:61:_:VAL	2.892	8.851	17	O	N	A:61:_:VAL		
A:58:_:LYS	HBOND:MC_MC	A:61:_:VAL	3.357	57.188	17	O	N	A:61:_:VAL		
A:58:_:LYS	HBOND:MC_MC	A:62:_:GLU	2.811	7.296	17	O	N	A:62:_:GLU		
A:58:_:LYS	VDW:SC_SC	A:62:_:GLU	3.663	-999.9	6	CE	CD			
A:59:_:GLU	HBOND:MC_MC	A:63:_:THR	2.941	9.465	17	O	N	A:63:_:THR		
A:60:_:GLN	HBOND:MC_MC	A:64:_:LEU	2.943	25.112	17	O	N	A:64:_:LEU		
A:61:_:VAL	HBOND:MC_MC	A:64:_:LEU	3.229	46.996	17	O	N	A:64:_:LEU		
A:61:_:VAL	HBOND:MC_MC	A:65:_:PHE	2.866	7.604	17	O	N	A:65:_:PHE		
A:62:_:GLU	HBOND:MC_MC	A:65:_:PHE	3.248	59.274	17	O	N	A:65:_:PHE		
A:62:_:GLU	HBOND:MC_MC	A:66:_:TYR	2.851	9.117	17	O	N	A:66:_:TYR		
A:62:_:GLU	VDW:SC_SC	A:83:_:LEU	3.852	-999.9	6	CD	CD1			
A:63:_:THR	VDW:MC_MC	A:66:_:TYR	3.842	-999.9	6	C	C			
A:63:_:THR	HBOND:MC_MC	A:67:_:LYS	3.003	54.065	17	O	N	A:67:_:LYS		
A:63:_:THR	HBOND:MC_MC	A:68:_:SER	3.36	19.246	17	O	N	A:68:_:SER		
A:64:_:LEU	HBOND:MC_MC	A:68:_:SER	3.236	47.755	17	O	N	A:68:_:SER		
A:64:_:LEU	VDW:SC_SC	A:68:_:SER	3.78	-999.9	6	CD1	CB			
A:64:_:LEU	HBOND:MC_MC	A:69:_:ILE	2.947	55.783	17	O	N	A:69:_:ILE		
A:64:_:LEU	VDW:SC_SC	A:69:_:ILE	3.854	-999.9	6	CG	CD1			
A:65:_:PHE	HBOND:MC_MC	A:69:_:ILE	3.283	25.645	17	O	N	A:69:_:ILE		
A:65:_:PHE	HBOND:MC_MC	A:70:_:LEU	3.006	20.992	17	O	N	A:70:_:LEU		
A:65:_:PHE	VDW:SC_SC	A:78:_:CYS	3.6	-999.9	6	CE2	SG			
A:65:_:PHE	VDW:SC_SC	A:80:_:ILE	3.381	-999.9	6	CZ	CG1			
A:66:_:TYR	VDW:SC_SC	A:70:_:LEU	3.564	-999.9	6	CD2	CD2			
A:66:_:TYR	HBOND:SC_SC	A:71:_:LYS	3.457	43.009	17	OH	NZ	A:71:_:LYS		
A:67:_:LYS	VDW:SC_SC	A:71:_:LYS	3.908	-999.9	6	CB	CD			
A:69:_:ILE	HBOND:MC_MC	A:72:_:ASP	2.975	15.283	17	O	N	A:72:_:ASP		
A:69:_:ILE	HBOND:MC_SC	A:75:_:HIS	2.849	1.894	17	O	NE2	A:75:_:HIS		
A:69:_:ILE	VDW:SC_SC	A:75:_:HIS	3.377	-999.9	6	CG2	CE1			
A:70:_:LEU	VDW:SC_SC	A:78:_:CYS	3.5	-999.9	6	CD1	CB			
A:72:_:ASP	VDW:SC_SC	A:75:_:HIS	3.988	-999.9	6	CB	CD2			
A:79:_:GLU	VDW:SC_SC	A:120:_:TYR	3.526	-999.9	6	CG	CE2			
A:81:_:ILE	VDW:SC_SC	A:127:_:ILE	3.414	-999.9	6	CG2	CD1			
A:89:_:ARG	VDW:SC_SC	A:92:_:LYS	4.035	-999.9	6	CB	CB			
A:91:_:PHE	HBOND:MC_MC	A:94:_:TRP	2.905	8.943	17	O	N	A:94:_:TRP		
A:91:_:PHE	VDW:SC_SC	A:94:_:TRP	3.961	-999.9	6	CD1	CE3			
A:91:_:PHE	VDW:SC_SC	A:131:_:ILE	3.609	-999.9	6	CE2	CG2			
A:91:_:PHE	VDW:SC_MC	A:134:_:LEU	4.022	-999.9	6	CE1	C			
A:91:_:PHE	VDW:SC_SC	A:135:_:VAL	3.261	-999.9	6	CZ	CG2			
A:94:_:TRP	VDW:SC_SC	A:97:_:LYS	3.568	-999.9	6	CZ2	CE			
A:94:_:TRP	VDW:SC_SC	A:139:_:ASN	3.773	-999.9	6	CD1	ND2			
A:97:_:LYS	HBOND:SC_MC	A:134:_:LEU	2.67	25.868	17	NZ	O	A:97:_:LYS		
A:97:_:LYS	HBOND:SC_MC	A:135:_:VAL	2.727	52.349	17	NZ	O	A:97:_:LYS		
A:97:_:LYS	HBOND:SC_MC	A:137:_:GLN	2.659	21.603	17	NZ	O	A:97:_:LYS		

A:97:_:LYS	VDW:SC_SC	A:139:_:ASN	3.366	-999.9	6	CG	ND2		
A:98:_:PHE	HBOND:MC_MC	A:140:_:LEU	3.072	8.944	17	O	N	A:140:_:LEU	
A:98:_:PHE	VDW:SC_SC	A:140:_:LEU	3.747	-999.9	6	CE2	CD2		
A:99:_:ALA	VDW:SC_SC	A:137:_:GLN	3.298	-999.9	6	CB	NE2		
A:100:_:PRO	VDW:SC_SC	A:137:_:GLN	3.352	-999.9	6	CD	OE1		
A:100:_:PRO	VDW:SC_SC	A:140:_:LEU	3.596	-999.9	6	CD	CB		
A:101:_:ILE	VDW:SC_SC	A:106:_:LYS	3.563	-999.9	6	CD1	CE		
A:102:_:ASN	HBOND:SC_MC	A:105:_:ILE	2.969	11.128	17	OD1	N	A:105:_:ILE	
A:102:_:ASN	VDW:SC_SC	A:105:_:ILE	3.586	-999.9	6	OD1	CG1		
A:102:_:ASN	HBOND:MC_MC	A:106:_:LYS	3.108	12.052	17	O	N	A:106:_:LYS	
A:102:_:ASN	HBOND:SC_SC	A:137:_:GLN	2.871	36.056	17	ND2	OE1	A:102:_:ASN	
A:102:_:ASN	VDW:SC_SC	A:137:_:GLN	3.191	-999.9	6	ND2	CD		
A:103:_:THR	HBOND:MC_MC	A:106:_:LYS	3.1	56.075	17	O	N	A:106:_:LYS	
A:103:_:THR	HBOND:MC_MC	A:107:_:ASP	2.853	6.713	17	O	N	A:107:_:ASP	
A:104:_:LYS	HBOND:MC_MC	A:108:_:PHE	3.053	14.418	17	O	N	A:108:_:PHE	
A:104:_:LYS	VDW:MC_SC	A:133:_:LEU	3.727	-999.9	6	C	CD2		
A:105:_:ILE	HBOND:MC_MC	A:108:_:PHE	3.302	52.428	17	O	N	A:108:_:PHE	
A:105:_:ILE	HBOND:MC_MC	A:109:_:PHE	3.202	7.437	17	O	N	A:109:_:PHE	
A:105:_:ILE	VDW:SC_SC	A:109:_:PHE	3.644	-999.9	6	CG2	CE2		
A:105:_:ILE	VDW:SC_SC	A:130:_:PHE	3.78	-999.9	6	CG2	CE1		
A:105:_:ILE	VDW:SC_SC	A:133:_:LEU	3.803	-999.9	6	CG1	CD2		
A:105:_:ILE	VDW:SC_SC	A:137:_:GLN	3.305	-999.9	6	CD1	NE2		
A:106:_:LYS	HBOND:MC_MC	A:109:_:PHE	3.324	54.717	17	O	N	A:109:_:PHE	
A:106:_:LYS	HBOND:MC_MC	A:110:_:PHE	2.875	8.244	17	O	N	A:110:_:PHE	
A:106:_:LYS	VDW:SC_SC	A:117:_:PHE	3.801	-999.9	6	CB	CE1		
A:107:_:ASP	HBOND:MC_MC	A:110:_:PHE	3.466	60.201	17	O	N	A:110:_:PHE	
A:107:_:ASP	HBOND:MC_MC	A:111:_:HIS	2.832	12.495	17	O	N	A:111:_:HIS	
A:108:_:PHE	HBOND:MC_MC	A:111:_:HIS	3.406	55.431	17	O	N	A:111:_:HIS	
A:108:_:PHE	HBOND:MC_MC	A:112:_:HIS	3.2	19.64	17	O	N	A:112:_:HIS	
A:108:_:PHE	PIPISTACK:SC_SC	A:112:_:HIS	5.196	117.861	9.4	7.156,-10.118,45.435	8.482,-15.052,46.385		N n1.52,p4.96
A:108:_:PHE	VDW:SC_SC	A:112:_:HIS	3.354	-999.9	6	CE1	CD2		
A:108:_:PHE	VDW:SC_SC	A:129:_:SER	3.471	-999.9	6	CE1	CB		
A:108:_:PHE	PIPISTACK:SC_SC	A:130:_:PHE	5.816	152.334	9.4	7.156,-10.118,45.435	5.428,-5.601,42.203		P n1.00,p7.28
A:108:_:PHE	VDW:SC_SC	A:130:_:PHE	3.569	-999.9	6	CE2	CD1		
A:108:_:PHE	VDW:SC_SC	A:133:_:LEU	3.28	-999.9	6	CB	CD2		
A:109:_:PHE	HBOND:MC_MC	A:112:_:HIS	3.447	43.755	17	O	N	A:112:_:HIS	
A:109:_:PHE	HBOND:MC_MC	A:113:_:HIS	3.148	58.95	17	O	N	A:113:_:HIS	
A:109:_:PHE	HBOND:MC_MC	A:114:_:VAL	2.796	6.943	17	O	N	A:114:_:VAL	
A:109:_:PHE	VDW:SC_SC	A:114:_:VAL	3.965	-999.9	6	CD1	CB		
A:109:_:PHE	PIPISTACK:SC_SC	A:117:_:PHE	5.947	105.216	9.4	10.202,-7.038,42.659	15.266,-4.503,44.477		N n0.39,p4.59
A:109:_:PHE	VDW:SC_SC	A:117:_:PHE	3.518	-999.9	6	CB	CD1		
A:109:_:PHE	VDW:SC_MC	A:118:_:ASN	3.961	-999.9	6	CE1	C		
A:109:_:PHE	VDW:SC_SC	A:122:_:LEU	3.773	-999.9	6	CE1	CD1		
A:109:_:PHE	PIPISTACK:SC_SC	A:130:_:PHE	5.007	135.05	9.4	10.202,-7.038,42.659	5.428,-5.601,42.203		T-FE n4.07,p3.24
A:109:_:PHE	VDW:SC_SC	A:130:_:PHE	3.424	-999.9	6	CE2	CE1		
A:110:_:PHE	HBOND:MC_MC	A:113:_:HIS	3.077	15.581	17	O	N	A:113:_:HIS	
A:114:_:VAL	HBOND:MC_MC	A:117:_:PHE	3.338	11.072	17	O	N	A:117:_:PHE	
A:118:_:ASN	HBOND:MC_MC	A:121:_:LEU	3.185	10.735	17	O	N	A:121:_:LEU	
A:119:_:PRO	HBOND:MC_MC	A:122:_:LEU	3.195	12.524	17	O	N	A:122:_:LEU	
A:119:_:PRO	VDW:SC_SC	A:130:_:PHE	3.63	-999.9	6	CG	CE2		
A:122:_:LEU	VDW:SC_SC	A:126:_:SER	4.016	-999.9	6	CD2	CB		
A:122:_:LEU	VDW:SC_SC	A:127:_:ILE	3.558	-999.9	6	CB	CG1		
A:122:_:LEU	VDW:SC_SC	A:130:_:PHE	3.603	-999.9	6	CD1	CB		
A:123:_:ASN	HBOND:SC_MC	A:126:_:SER	2.985	23.184	17	OD1	N	A:126:_:SER	
A:123:_:ASN	HBOND:MC_MC	A:127:_:ILE	2.945	10.098	17	O	N	A:127:_:ILE	
A:123:_:ASN	VDW:MC_SC	A:127:_:ILE	3.826	-999.9	6	C	CD1		
A:124:_:THR	VDW:MC_SC	A:128:_:PRO	3.765	-999.9	6	C	CD		
A:125:_:ASN	VDW:MC_SC	A:128:_:PRO	3.718	-999.9	6	C	CD		
A:125:_:ASN	HBOND:MC_SC	A:129:_:SER	3.409	43.581	17	O	OG	A:129:_:SER	
A:126:_:SER	HBOND:MC_MC	A:129:_:SER	3.458	58.399	17	O	N	A:129:_:SER	
A:126:_:SER	HBOND:MC_MC	A:130:_:PHE	2.889	13.486	17	O	N	A:130:_:PHE	
A:127:_:ILE	HBOND:MC_MC	A:130:_:PHE	3.147	56.13	17	O	N	A:130:_:PHE	
A:127:_:ILE	HBOND:MC_MC	A:131:_:ILE	2.814	14.079	17	O	N	A:131:_:ILE	
A:128:_:PRO	HBOND:MC_MC	A:131:_:ILE	3.121	57.061	17	O	N	A:131:_:ILE	
A:128:_:PRO	VDW:MC_SC	A:131:_:ILE	4.003	-999.9	6	C	CB		
A:128:_:PRO	HBOND:MC_MC	A:132:_:GLU	2.874	15.733	17	O	N	A:132:_:GLU	
A:129:_:SER	HBOND:MC_MC	A:132:_:GLU	3.321	53.848	17	O	N	A:132:_:GLU	
A:129:_:SER	HBOND:MC_MC	A:133:_:LEU	3.179	10.302	17	O	N	A:133:_:LEU	
A:130:_:PHE	HBOND:MC_MC	A:133:_:LEU	3.281	55.356	17	O	N	A:133:_:LEU	
A:130:_:PHE	HBOND:MC_MC	A:134:_:LEU	3.036	14.84	17	O	N	A:134:_:LEU	
A:130:_:PHE	VDW:SC_SC	A:134:_:LEU	3.736	-999.9	6	CE2	CD1		
A:131:_:ILE	HBOND:MC_MC	A:134:_:LEU	3.082	52.751	17	O	N	A:134:_:LEU	
A:131:_:ILE	HBOND:MC_MC	A:135:_:VAL	2.819	17.11	17	O	N	A:135:_:VAL	
A:132:_:GLU	HBOND:MC_MC	A:135:_:VAL	3.128	52.63	17	O	N	A:135:_:VAL	
A:132:_:GLU	HBOND:MC_MC	A:136:_:ASP	2.965	17.566	17	O	N	A:136:_:ASP	
A:133:_:LEU	HBOND:MC_MC	A:137:_:GLN	3.03	26.845	17	O	N	A:137:_:GLN	
A:134:_:LEU	VDW:SC_SC	A:137:_:GLN	3.646	-999.9	6	CD2	NE2		
**LIGAND INTERACTIONS**									
_:1:_:FMN	IAC:LIG_SC	A:8:_:TYR	2.92	-999.9	0	C6	HH		
_:1:_:FMN	IAC:LIG_SC	A:10:_:SER	4.732	-999.9	0	C7M	OG		
_:1:_:FMN	IAC:LIG_SC	A:22:_:ASP	6.504	-999.9	0	C8M	HB3		
_:1:_:FMN	IAC:LIG_SC	A:23:_:LEU	6.46	-999.9	0	C7M	HD22		
_:1:_:FMN	IAC:LIG_MC	A:25:_:ASP	6.458	-999.9	0	O3'	O		
_:1:_:FMN	VDW:LIG_SC	A:26:_:ILE	3.747	-999.9	6	C8	CG2		
_:1:_:FMN	IAC:LIG_SC	A:27:_:LEU	6.348	-999.9	0	N5	HD22		
_:1:_:FMN	IAC:LIG_SC	A:29:_:GLU	2.663	-999.9	0	O3'	OE1		
_:1:_:FMN	VDW:LIG_SC	A:30:_:ALA	3.347	-999.9	6	C4	CB		
_:1:_:FMN	IAC:LIG_MC	A:31:_:VAL	5.539	-999.9	0	N3	N		
_:1:_:FMN	IAC:LIG_MC	A:32:_:LYS	6.145	-999.9	0	O2	C		
_:1:_:FMN	IAC:LIG_SC	A:33:_:PHE	2.256	-999.9	0	O2	HB3		
_:1:_:FMN	IAC:LIG_SC	A:34:_:ASN	2.245	-999.9	0	O4	HD22		
_:1:_:FMN	IAC:LIG_SC	A:35:_:SER	6.995	-999.9	0	O2	H		
_:1:_:FMN	IAC:LIG_SC	A:37:_:ASN	5.882	-999.9	0	O2	HD21		
_:1:_:FMN	IAC:LIG_SC	A:39:_:ILE	4.444	-999.9	0	O4	HG21		
_:1:_:FMN	IAC:LIG_MC	A:40:_:THR	6.906	-999.9	0	O4	O		
_:1:_:FMN	IAC:LIG_SC	A:41:_:GLY	6.5	-999.9	0	O4	HA1		
_:1:_:FMN	IAC:LIG_SC	A:43:_:LEU	3.181	-999.9	0	C7M	HD11		
_:1:_:FMN	IAC:LIG_MC	A:44:_:TYR	6.971	-999.9	0	C7M	O		
_:1:_:FMN	IAC:LIG_SC	A:45:_:TYR	6.556	-999.9	0	C7M	HD1		
_:1:_:FMN	IAC:LIG_SC	A:50:_:PHE	3.74	-999.9	0	C7M	CG		
_:1:_:FMN	IAC:LIG_MC	A:51:_:LEU	6.527	-999.9	0	C7M	N		

_:1:_:FMN	VDW:LIG_SC	A:52:_:GLN	3.655	-999.9	6	C6	CD
_:1:_:FMN	IAC:LIG_MC	A:53:_:TYR	6.5	-999.9	0	O4	O
_:1:_:FMN	IAC:LIG_SC	A:54:_:LEU	2.812	-999.9	0	O4	HD23
_:1:_:FMN	VDW:LIG_SC	A:64:_:LEU	3.308	-999.9	6	C2	CD1
_:1:_:FMN	IAC:LIG_SC	A:67:_:LYS	2.519	-999.9	0	O2P	HZ2
_:1:_:FMN	IAC:LIG_MC	A:68:_:SER	2.283	-999.9	0	O2'	O
_:1:_:FMN	IAC:LIG_SC	A:69:_:ILE	2.66	-999.9	0	O2'	HA
_:1:_:FMN	IAC:LIG_MC	A:70:_:LEU	5.733	-999.9	0	O2'	N
_:1:_:FMN	IAC:LIG_SC	A:71:_:LYS	5.069	-999.9	0	O4'	HB2
_:1:_:FMN	IAC:LIG_SC	A:72:_:ASP	2.694	-999.9	0	O2'	HB2
_:1:_:FMN	IAC:LIG_SC	A:73:_:SER	5.621	-999.9	0	O4'	H
_:1:_:FMN	IAC:LIG_SC	A:74:_:ARG	3.152	-999.9	0	C1'	HH21
_:1:_:FMN	IAC:LIG_SC	A:75:_:HIS	3.619	-999.9	0	C7M	ND1
_:1:_:FMN	IAC:LIG_MC	A:95:_:SER	5.481	-999.9	0	O4	O
_:1:_:FMN	IAC:LIG_SC	A:96:_:MET	2.83	-999.9	0	O4	HE1

NodeId1	Interaction	NodeId2	Distance(*)	Angle	Energy	Atom1	Atom2	Donor	Positive	Cation	Orientation
AnBLUF65 LIGHT											
A:1:_:MET	HBOND:MC_MC	A:57:_:LYS	3.051	26.757	17	O	N	A:57:_:LYS			
A:1:_:MET	VDW:SC_SC	A:83:_:SER	3.771	-999.9	6	CG	CB				
A:2:_:ASN	VDW:SC_MC	A:55:_:GLY	3.663	-999.9	6	OD1	C				
A:2:_:ASN	HBOND:MC_MC	A:84:_:ILE	3.24	17.071	17	O	N	A:84:_:ILE			
A:2:_:ASN	VDW:MC_SC	A:84:_:ILE	3.828	-999.9	6	C	CG1				
A:3:_:VAL	HBOND:MC_MC	A:55:_:GLY	2.829	7.497	17	N	O	A:3:_:VAL			
A:3:_:VAL	VDW:SC_SC	A:57:_:LYS	3.929	-999.9	6	CG1	CB				
A:3:_:VAL	VDW:MC_SC	A:84:_:ILE	3.694	-999.9	6	C	CD1				
A:4:_:ARG	VDW:SC_SC	A:52:_:CYS	3.501	-999.9	6	CD	SG				
A:4:_:ARG	HBOND:SC_SC	A:54:_:GLU	2.889	30.3	17	NE	OE2	A:4:_:ARG			
A:4:_:ARG	HBOND:MC_MC	A:82:_:TYR	2.873	6.851	17	N	O	A:4:_:ARG			
A:4:_:ARG	VDW:SC_SC	A:84:_:ILE	3.641	-999.9	6	CB	CG2				
A:4:_:ARG	HBOND:SC_SC	A:88:_:SER	3.053	18.415	17	NH2	OG	A:4:_:ARG			
A:4:_:ARG	VDW:SC_SC	A:129:_:LEU	3.584	-999.9	6	CD	CD1				
A:5:_:LEU	HBOND:MC_MC	A:53:_:LEU	2.83	7.704	17	O	N	A:53:_:LEU			
A:5:_:LEU	VDW:SC_SC	A:60:_:VAL	3.62	-999.9	6	CB	CG1				
A:5:_:LEU	VDW:SC_SC	A:64:_:PHE	3.737	-999.9	6	CD2	CG				
A:5:_:LEU	VDW:SC_SC	A:78:_:TRP	4.036	-999.9	6	CG	CD1				
A:6:_:CYS	VDW:SC_SC	A:50:_:PHE	3.87	-999.9	6	CB	CZ				
A:6:_:CYS	VDW:SC_SC	A:52:_:CYS	3.753	-999.9	6	SG	CB				
A:6:_:CYS	HBOND:MC_MC	A:79:_:LEU	2.905	16.641	17	O	N	A:79:_:LEU			
A:6:_:CYS	HBOND:MC_MC	A:80:_:CYS	2.84	8.351	17	N	O	A:6:_:CYS			
A:6:_:CYS	VDW:SC_SC	A:80:_:CYS	3.654	-999.9	6	CB	SG				
A:6:_:CYS	VDW:SC_SC	A:129:_:LEU	4.13	-999.9	6	SG	CD1				
A:7:_:TYR	HBOND:MC_MC	A:51:_:GLN	3.004	8.983	17	O	N	A:51:_:GLN			
A:7:_:TYR	VDW:SC_SC	A:51:_:GLN	3.273	-999.9	6	CE2	OE1				
A:7:_:TYR	PIPISTACK:SC_SC	A:64:_:PHE	5.227	145.862	9.4	0.841,-0.482,30.992	-1.941,-2.407,27.007				T-FE n4.02,p2.89
A:7:_:TYR	VDW:SC_SC	A:64:_:PHE	3.483	-999.9	6	CD2	CE1				
A:7:_:TYR	VDW:SC_SC	A:67:_:ILE	3.738	-999.9	6	CZ	CG2				
A:7:_:TYR	PIPISTACK:SC_SC	A:73:_:HIS	5.997	107.606	9.4	0.841,-0.482,30.992	3.535,4.418,28.827				N n1.31,p6.21
A:7:_:TYR	VDW:SC_SC	A:73:_:HIS	3.798	-999.9	6	CE1	CE1				
A:7:_:TYR	VDW:SC_SC	A:76:_:VAL	3.715	-999.9	6	CD1	CG1				
A:7:_:TYR	VDW:SC_MC	A:77:_:LYS	3.908	-999.9	6	CB	C				
A:7:_:TYR	VDW:MC_SC	A:79:_:LEU	3.872	-999.9	6	C	CD1				
A:8:_:ALA	HBOND:MC_MC	A:77:_:LYS	2.768	4.3	17	O	N	A:77:_:LYS			
A:8:_:ALA	VDW:SC_SC	A:118:_:ILE	3.258	-999.9	6	CB	CG1				
A:9:_:SER	HBOND:MC_MC	A:49:_:PHE	2.937	18.684	17	O	N	A:49:_:PHE			
A:9:_:SER	VDW:SC_SC	A:73:_:HIS	4.032	-999.9	6	CB	CG				
A:9:_:SER	VDW:SC_SC	A:76:_:VAL	3.967	-999.9	6	CB	CG2				
A:10:_:GLN	HBOND:MC_MC	A:74:_:HIS	2.824	9.432	17	N	O	A:10:_:GLN			
A:10:_:GLN	VDW:SC_SC	A:74:_:HIS	4.001	-999.9	6	CB	CE1				
A:10:_:GLN	HBOND:SC_SC	A:75:_:ASN	2.964	28.036	17	OE1	ND2	A:75:_:ASN			
A:10:_:GLN	VDW:SC_SC	A:75:_:ASN	3.442	-999.9	6	OE1	CG				
A:11:_:ARG	VDW:SC_SC	A:18:_:LEU	3.599	-999.9	6	CD	CD1				
A:11:_:ARG	HBOND:SC_SC	A:44:_:TYR	3.341	51.286	17	NE	OH	A:11:_:ARG			
A:11:_:ARG	HBOND:MC_MC	A:47:_:ASN	2.757	12.845	17	N	O	A:11:_:ARG			
A:11:_:ARG	VDW:SC_SC	A:47:_:ASN	3.284	-999.9	6	CZ	OD1				
A:11:_:ARG	VDW:MC_SC	A:74:_:HIS	3.806	-999.9	6	C	CE1				
A:12:_:ASN	HBOND:MC_MC	A:15:_:ASN	3.291	18.503	17	O	N	A:15:_:ASN			
A:12:_:ASN	VDW:SC_SC	A:15:_:ASN	3.688	-999.9	6	CB	OD1				
A:12:_:ASN	HBOND:MC_MC	A:72:_:ARG	3.355	13.049	17	N	O	A:12:_:ASN			
A:17:_:ASP	HBOND:SC_MC	A:20:_:GLN	3.05	11.722	17	OD1	N	A:20:_:GLN			
A:17:_:ASP	VDW:SC_SC	A:20:_:GLN	3.476	-999.9	6	CG	CB				
A:17:_:ASP	HBOND:MC_MC	A:21:_:ASP	2.966	7.333	17	O	N	A:21:_:ASP			
A:18:_:LEU	HBOND:MC_MC	A:21:_:ASP	3.252	61.488	17	O	N	A:21:_:ASP			
A:18:_:LEU	HBOND:MC_MC	A:22:_:LEU	2.86	7.95	17	O	N	A:22:_:LEU			
A:18:_:LEU	VDW:SC_SC	A:22:_:LEU	3.701	-999.9	6	CD1	CG				
A:18:_:LEU	VDW:SC_SC	A:44:_:TYR	3.642	-999.9	6	CD2	CE2				
A:19:_:LEU	HBOND:MC_MC	A:23:_:ARG	3.219	11.474	17	O	N	A:23:_:ARG			
A:19:_:LEU	VDW:SC_SC	A:23:_:ARG	3.963	-999.9	6	CD1	CZ				
A:20:_:GLN	HBOND:MC_MC	A:23:_:ARG	3.068	55.16	17	O	N	A:23:_:ARG			
A:20:_:GLN	HBOND:MC_MC	A:24:_:ASP	2.84	8.256	17	O	N	A:24:_:ASP			
A:20:_:GLN	VDW:SC_SC	A:24:_:ASP	3.539	-999.9	6	NE2	CG				
A:21:_:ASP	HBOND:MC_MC	A:24:_:ASP	3.335	57.756	17	O	N	A:24:_:ASP			
A:21:_:ASP	HBOND:MC_MC	A:25:_:ILE	2.851	6.66	17	O	N	A:25:_:ILE			
A:21:_:ASP	VDW:MC_SC	A:25:_:ILE	3.909	-999.9	6	C	CD1				
A:21:_:ASP	VDW:SC_SC	A:72:_:ARG	3.883	-999.9	6	CG	CD				
A:22:_:LEU	HBOND:MC_MC	A:26:_:LEU	3.029	9.373	17	O	N	A:26:_:LEU			
A:22:_:LEU	VDW:SC_SC	A:49:_:PHE	3.65	-999.9	6	CD2	CE1				
A:23:_:ARG	HBOND:MC_MC	A:26:_:LEU	3.413	56.695	17	O	N	A:26:_:LEU			
A:23:_:ARG	HBOND:MC_MC	A:27:_:THR	2.888	8.146	17	O	N	A:27:_:THR			
A:23:_:ARG	VDW:SC_SC	A:140:_:VAL	3.399	-999.9	6	CG	CG1				
A:24:_:ASP	HBOND:MC_MC	A:27:_:THR	3.262	58.143	17	O	N	A:27:_:THR			
A:24:_:ASP	HBOND:MC_MC	A:28:_:GLU	2.899	1.341	17	O	N	A:28:_:GLU			
A:24:_:ASP	VDW:MC_SC	A:28:_:GLU	3.937	-999.9	6	C	CD				
A:25:_:ILE	HBOND:MC_MC	A:29:_:ALA	2.883	14.173	17	O	N	A:29:_:ALA			
A:25:_:ILE	VDW:SC_SC	A:42:_:LEU	3.854	-999.9	6	CG2	CD2				
A:25:_:ILE	VDW:SC_SC	A:49:_:PHE	4.011	-999.9	6	CD1	CZ				
A:25:_:ILE	VDW:SC_SC	A:72:_:ARG	3.656	-999.9	6	CG1	CZ				
A:26:_:LEU	HBOND:MC_MC	A:30:_:ARG	2.899	18.67	17	O	N	A:30:_:ARG			
A:26:_:LEU	VDW:SC_SC	A:42:_:LEU	3.992	-999.9	6	CD2	CD2				
A:26:_:LEU	VDW:SC_SC	A:96:_:TYR	3.653	-999.9	6	CD1	CG				
A:26:_:LEU	VDW:SC_SC	A:140:_:VAL	3.542	-999.9	6	CD1	CG2				
A:27:_:THR	HBOND:MC_MC	A:30:_:ARG	3.361	45.526	17	O	N	A:30:_:ARG			
A:27:_:THR	HBOND:MC_MC	A:31:_:ASP	3.082	3.821	17	O	N	A:31:_:ASP			
A:28:_:GLU	HBOND:MC_MC	A:32:_:PHE	3.094	7.187	17	O	N	A:32:_:PHE			
A:28:_:GLU	IONIC:SC_SC	A:72:_:ARG	3.49	138.132	20	-2.364,13.251,31.342	CZ		A:72:_:ARG		
A:28:_:GLU	VDW:SC_SC	A:72:_:ARG	3.911	-999.9	6	CD	CZ				
A:29:_:ALA	HBOND:MC_MC	A:33:_:ASN	2.882	14.398	17	O	N	A:33:_:ASN			
A:29:_:ALA	VDW:MC_SC	A:33:_:ASN	3.967	-999.9	6	C	CG				
A:30:_:ARG	HBOND:MC_MC	A:34:_:ASP	3.141	12.47	17	O	N	A:34:_:ASP			
A:30:_:ARG	HBOND:SC_MC	A:94:_:MET	2.835	15.072	17	NE	O	A:30:_:ARG			
A:30:_:ARG	HBOND:SC_SC	A:137:_:GLN	3.005	9.749	17	NH2	OE1	A:30:_:ARG			
A:31:_:ASP	HBOND:MC_MC	A:35:_:LEU	3.371	16.093	17	O	N	A:35:_:LEU			
A:32:_:PHE	HBOND:MC_MC	A:35:_:LEU	3.25	49.604	17	O	N	A:35:_:LEU			
A:32:_:PHE	HBOND:MC_MC	A:36:_:ASN	3.064	46.348	17	O	N	A:36:_:ASN			
A:32:_:PHE	VDW:SC_SC	A:36:_:ASN	3.396	-999.9	6	CE1	ND2				
A:32:_:PHE	PICATION:SC_SC	A:66:_:LYS	3.424	172.065	9.6	CE1	NZ			A:66:_:LYS	
A:33:_:ASN	HBOND:MC_MC	A:36:_:ASN	3.298	22.45	17	O	N	A:36:_:ASN			
A:33:_:ASN	HBOND:MC_MC	A:37:_:GLU	3.077	55.694	17	O	N	A:37:_:GLU			
A:33:_:ASN	HBOND:MC_MC	A:38:_:ILE	2.897	4.07	17	O	N	A:38:_:ILE			

A:33:_:ASN	VDW:SC_SC	A:38:_:ILE	3.76	-999.9	6	CG	CG2		
A:33:_:ASN	VDW:SC_SC	A:63:_:LEU	3.451	-999.9	6	OD1	CD2		
A:33:_:ASN	VDW:SC_MC	A:93:_:SER	4.016	-999.9	6	CB	C		
A:33:_:ASN	VDW:SC_SC	A:94:_:MET	3.39	-999.9	6	ND2	CE		
A:38:_:ILE	VDW:SC_SC	A:59:_:VAL	3.925	-999.9	6	CD1	CG2		
A:38:_:ILE	VDW:SC_SC	A:63:_:LEU	3.654	-999.9	6	CD1	CD2		
A:39:_:CYS	HBOND:MC_MC	A:54:_:GLU	2.862	6.375	17	O	N	A:54:_:GLU	
A:39:_:CYS	VDW:SC_SC	A:54:_:GLU	3.708	-999.9	6	SG	CB		
A:39:_:CYS	VDW:SC_MC	A:89:_:PHE	3.881	-999.9	6	SG	C		
A:40:_:GLY	VDW:MC_SC	A:89:_:PHE	3.856	-999.9	6	C	CB		
A:40:_:GLY	HBOND:MC_MC	A:92:_:TRP	3.169	27.921	17	N	O	A:40:_:GLY	
A:40:_:GLY	VDW:MC_SC	A:94:_:MET	3.755	-999.9	6	C	SD		
A:40:_:GLY	HBOND:MC_MC	A:95:_:LYS	2.881	7.699	17	O	N	A:95:_:LYS	
A:41:_:VAL	HBOND:MC_MC	A:52:_:CYS	2.947	7.612	17	O	N	A:52:_:CYS	
A:41:_:VAL	VDW:SC_SC	A:52:_:CYS	3.866	-999.9	6	CG1	CB		
A:41:_:VAL	VDW:SC_SC	A:89:_:PHE	3.581	-999.9	6	CG1	CE1		
A:41:_:VAL	VDW:MC_SC	A:94:_:MET	3.408	-999.9	6	C	SD		
A:41:_:VAL	VDW:SC_SC	A:95:_:LYS	3.868	-999.9	6	CG2	CB		
A:41:_:VAL	VDW:SC_SC	A:97:_:VAL	3.683	-999.9	6	CG2	CG2		
A:41:_:VAL	VDW:SC_SC	A:132:_:LEU	3.931	-999.9	6	CG1	CD1		
A:42:_:LEU	VDW:SC_SC	A:49:_:PHE	3.804	-999.9	6	CD1	CD1		
A:42:_:LEU	VDW:SC_SC	A:51:_:GLN	3.87	-999.9	6	CD1	CB		
A:42:_:LEU	VDW:SC_SC	A:94:_:MET	3.795	-999.9	6	CB	SD		
A:42:_:LEU	HBOND:MC_MC	A:95:_:LYS	2.769	4.649	17	N	O	A:42:_:LEU	
A:42:_:LEU	HBOND:MC_MC	A:97:_:VAL	2.977	10.312	17	O	N	A:97:_:VAL	
A:43:_:TYR	HBOND:MC_MC	A:50:_:PHE	2.742	5.821	17	N	O	A:43:_:TYR	
A:43:_:TYR	VDW:SC_MC	A:97:_:VAL	4.029	-999.9	6	CD1	C		
A:43:_:TYR	VDW:SC_MC	A:98:_:GLN	3.693	-999.9	6	CE1	C		
A:43:_:TYR	VDW:SC_SC	A:103:_:ILE	3.873	-999.9	6	CE1	CD1		
A:43:_:TYR	PIPISTACK:SC_SC	A:128:_:PHE	5.321	91.4	9.4	6.739,-0.629,41.835	5.193,-5.719,41.692		N n5.20,p0.51
A:43:_:TYR	VDW:SC_SC	A:128:_:PHE	3.821	-999.9	6	CD2	CZ		
A:43:_:TYR	VDW:SC_SC	A:132:_:LEU	3.628	-999.9	6	CD1	CD2		
A:44:_:TYR	HBOND:SC_MC	A:47:_:ASN	3.372	43.208	17	OH	N	A:47:_:ASN	
A:44:_:TYR	VDW:SC_MC	A:47:_:ASN	4.023	-999.9	6	CE1	C		
A:44:_:TYR	VDW:SC_MC	A:48:_:ALA	3.905	-999.9	6	CD1	C		
A:44:_:TYR	PIPISTACK:SC_SC	A:49:_:PHE	5.059	124.419	9.4	9.013,6.081,38.083	5.647,5.464,34.357		T-FE n4.68,p1.82
A:44:_:TYR	VDW:SC_SC	A:49:_:PHE	3.562	-999.9	6	CD1	CE1		
A:44:_:TYR	VDW:SC_SC	A:96:_:TYR	3.905	-999.9	6	CB	CE1		
A:44:_:TYR	HBOND:MC_MC	A:97:_:VAL	3.148	15.454	17	N	O	A:44:_:TYR	
A:45:_:ALA	HBOND:MC_MC	A:48:_:ALA	2.95	27.086	17	O	N	A:48:_:ALA	
A:45:_:ALA	VDW:SC_SC	A:99:_:ARG	3.818	-999.9	6	CB	CB		
A:49:_:PHE	PIPISTACK:SC_SC	A:73:_:HIS	6.011	97.333	9.4	5.647,5.464,34.357	3.535,4.418,28.827		L n4.45,p4.01
A:49:_:PHE	VDW:SC_SC	A:73:_:HIS	3.925	-999.9	6	CD2	CB		
A:50:_:PHE	VDW:SC_SC	A:79:_:LEU	3.635	-999.9	6	CE2	CD1		
A:50:_:PHE	VDW:SC_SC	A:117:_:PRO	3.652	-999.9	6	CD2	CB		
A:50:_:PHE	VDW:SC_SC	A:125:_:LEU	3.754	-999.9	6	CE2	CD1		
A:50:_:PHE	PIPISTACK:SC_SC	A:128:_:PHE	5.175	103.804	9.4	2.581,-4.461,37.406	5.193,-5.719,41.692		L n5.00,p3.65
A:50:_:PHE	VDW:SC_SC	A:128:_:PHE	3.669	-999.9	6	CD1	CE2		
A:50:_:PHE	VDW:SC_SC	A:129:_:LEU	3.739	-999.9	6	CE1	CD2		
A:50:_:PHE	VDW:SC_SC	A:132:_:LEU	3.737	-999.9	6	CD1	CD1		
A:51:_:GLN	VDW:SC_SC	A:67:_:ILE	3.442	-999.9	6	OE1	CD1		
A:51:_:GLN	VDW:SC_SC	A:94:_:MET	3.328	-999.9	6	NE2	CE		
A:52:_:CYS	VDW:SC_SC	A:89:_:PHE	3.927	-999.9	6	SG	CD2		
A:52:_:CYS	VDW:SC_SC	A:129:_:LEU	3.771	-999.9	6	CB	CD2		
A:53:_:LEU	VDW:SC_SC	A:60:_:VAL	3.849	-999.9	6	CB	CG1		
A:53:_:LEU	VDW:SC_SC	A:63:_:LEU	3.944	-999.9	6	CD2	CD2		
A:53:_:LEU	VDW:SC_SC	A:94:_:MET	3.692	-999.9	6	CD2	CE		
A:54:_:GLU	VDW:MC_SC	A:60:_:VAL	3.975	-999.9	6	C	CG2		
A:54:_:GLU	HBOND:SC_MC	A:88:_:SER	3.311	31.156	17	OE2	N	A:88:_:SER	
A:54:_:GLU	HBOND:SC_MC	A:89:_:PHE	2.783	7.8	17	OE1	N	A:89:_:PHE	
A:54:_:GLU	VDW:SC_SC	A:89:_:PHE	3.851	-999.9	6	CD	CD2		
A:55:_:GLY	VDW:MC_SC	A:60:_:VAL	3.692	-999.9	6	C	CG2		
A:56:_:GLU	HBOND:SC_MC	A:59:_:VAL	3.062	20.944	17	OE1	N	A:59:_:VAL	
A:56:_:GLU	VDW:SC_SC	A:59:_:VAL	3.937	-999.9	6	CB	CG1		
A:56:_:GLU	HBOND:MC_MC	A:60:_:VAL	3.007	9.32	17	O	N	A:60:_:VAL	
A:57:_:LYS	HBOND:MC_MC	A:60:_:VAL	3.397	54.915	17	O	N	A:60:_:VAL	
A:57:_:LYS	HBOND:MC_MC	A:61:_:GLU	2.898	7.822	17	O	N	A:61:_:GLU	
A:57:_:LYS	VDW:SC_SC	A:61:_:GLU	3.674	-999.9	6	CE	CD		
A:58:_:GLU	HBOND:MC_MC	A:62:_:ARG	2.968	8.208	17	O	N	A:62:_:ARG	
A:58:_:GLU	VDW:MC_SC	A:62:_:ARG	3.689	-999.9	6	C	CZ		
A:59:_:VAL	HBOND:MC_MC	A:63:_:LEU	3.03	16.127	17	O	N	A:63:_:LEU	
A:60:_:VAL	HBOND:MC_MC	A:63:_:LEU	3.268	54.264	17	O	N	A:63:_:LEU	
A:60:_:VAL	HBOND:MC_MC	A:64:_:PHE	2.895	11.683	17	O	N	A:64:_:PHE	
A:61:_:GLU	HBOND:MC_MC	A:64:_:PHE	3.275	55.698	17	O	N	A:64:_:PHE	
A:61:_:GLU	HBOND:MC_MC	A:65:_:GLU	3.049	9.794	17	O	N	A:65:_:GLU	
A:62:_:ARG	HBOND:MC_MC	A:66:_:LYS	3.228	17.05	17	O	N	A:66:_:LYS	
A:63:_:LEU	HBOND:MC_MC	A:66:_:LYS	3.148	50.503	17	O	N	A:66:_:LYS	
A:63:_:LEU	VDW:SC_SC	A:66:_:LYS	3.647	-999.9	6	CD1	CE		
A:63:_:LEU	HBOND:MC_MC	A:67:_:ILE	2.903	11.868	17	O	N	A:67:_:ILE	
A:63:_:LEU	VDW:SC_SC	A:67:_:ILE	3.728	-999.9	6	CG	CD1		
A:64:_:PHE	HBOND:MC_MC	A:67:_:ILE	3.282	55.657	17	O	N	A:67:_:ILE	
A:64:_:PHE	HBOND:MC_MC	A:68:_:GLN	2.971	17	17	O	N	A:68:_:GLN	
A:64:_:PHE	VDW:SC_SC	A:68:_:GLN	3.083	-999.9	6	CE2	NE2		
A:64:_:PHE	VDW:SC_SC	A:76:_:VAL	3.658	-999.9	6	CE1	CG1		
A:64:_:PHE	VDW:SC_SC	A:78:_:TRP	4.005	-999.9	6	CE2	CD1		
A:65:_:GLU	HBOND:MC_SC	A:69:_:LYS	3.357	60.819	17	O	NZ	A:69:_:LYS	
A:66:_:LYS	HBOND:MC_MC	A:69:_:LYS	3.181	25.213	17	O	N	A:69:_:LYS	
A:67:_:ILE	HBOND:MC_MC	A:70:_:ASP	3.05	14.284	17	O	N	A:70:_:ASP	
A:67:_:ILE	HBOND:MC_SC	A:73:_:HIS	2.768	3.779	17	O	NE2	A:73:_:HIS	
A:67:_:ILE	VDW:SC_SC	A:73:_:HIS	3.406	-999.9	6	CG2	CE1		
A:73:_:HIS	VDW:SC_SC	A:76:_:VAL	3.932	-999.9	6	CE1	CG2		
A:78:_:TRP	HBOND:SC_SC	A:81:_:THR	2.85	24.255	17	NE1	OG1	A:78:_:TRP	
A:80:_:CYS	VDW:SC_SC	A:125:_:LEU	3.656	-999.9	6	SG	CD2		
A:84:_:ILE	VDW:SC_SC	A:87:_:ASN	3.378	-999.9	6	CD1	OD1		
A:87:_:ASN	VDW:MC_SC	A:90:_:GLN	3.831	-999.9	6	C	NE2		
A:89:_:PHE	HBOND:MC_MC	A:92:_:TRP	2.961	14.111	17	O	N	A:92:_:TRP	
A:89:_:PHE	VDW:SC_SC	A:92:_:TRP	3.934	-999.9	6	CD1	CZ3		
A:89:_:PHE	VDW:SC_MC	A:132:_:LEU	4.032	-999.9	6	CE1	C		
A:89:_:PHE	VDW:SC_SC	A:133:_:LEU	3.916	-999.9	6	CZ	CB		
A:92:_:TRP	VDW:SC_SC	A:95:_:LYS	3.513	-999.9	6	CZ2	CE		
A:92:_:TRP	VDW:SC_SC	A:133:_:LEU	3.753	-999.9	6	CZ3	CD1		
A:92:_:TRP	VDW:SC_SC	A:137:_:GLN	3.689	-999.9	6	CD1	NE2		
A:95:_:LYS	HBOND:SC_MC	A:132:_:LEU	2.678	29.553	17	NZ	O	A:95:_:LYS	

A:95:_:LYS	HBOND:SC_MC	A:133:_:LEU	2.804	52.309	17	NZ	O	A:95:_:LYS	
A:95:_:LYS	HBOND:SC_MC	A:135:_:ALA	2.665	20.525	17	NZ	O	A:95:_:LYS	
A:95:_:LYS	VDW:MC_SC	A:137:_:GLN	3.593	-999.9	6	C	OE1		
A:96:_:TYR	HBOND:MC_SC	A:137:_:GLN	2.979	26.403	17	N	OE1	A:96:_:TYR	
A:96:_:TYR	HBOND:MC_MC	A:138:_:THR	2.916	13.767	17	O	N	A:138:_:THR	
A:96:_:TYR	VDW:SC_SC	A:138:_:THR	3.641	-999.9	6	CD2	CB		
A:96:_:TYR	VDW:SC_SC	A:140:_:VAL	3.283	-999.9	6	CD2	CG2		
A:97:_:VAL	VDW:SC_SC	A:103:_:ILE	3.884	-999.9	6	CG1	CD1		
A:97:_:VAL	VDW:SC_SC	A:132:_:LEU	3.755	-999.9	6	CG1	CD2		
A:97:_:VAL	VDW:SC_SC	A:135:_:ALA	3.811	-999.9	6	CG1	CB		
A:98:_:GLN	VDW:SC_SC	A:138:_:THR	3.549	-999.9	6	OE1	CG2		
A:99:_:ARG	HBOND:SC_SC	A:104:_:GLU	2.567	38.95	17	NE	OE2	A:99:_:ARG	
A:99:_:ARG	VDW:SC_SC	A:104:_:GLU	3.89	-999.9	6	CG	CG		
A:99:_:ARG	VDW:SC_SC	A:115:_:PHE	4.025	-999.9	6	CZ	CD2		
A:100:_:ASN	HBOND:SC_MC	A:103:_:ILE	2.935	8.163	17	OD1	N	A:103:_:ILE	
A:100:_:ASN	VDW:SC_SC	A:103:_:ILE	3.508	-999.9	6	OD1	CG1		
A:100:_:ASN	HBOND:MC_MC	A:104:_:GLU	3.015	9.201	17	O	N	A:104:_:GLU	
A:101:_:THR	HBOND:MC_MC	A:104:_:GLU	3.456	60.576	17	O	N	A:104:_:GLU	
A:101:_:THR	HBOND:MC_MC	A:105:_:ALA	2.819	14.271	17	O	N	A:105:_:ALA	
A:102:_:ASN	HBOND:MC_MC	A:105:_:ALA	3.253	57.111	17	O	N	A:105:_:ALA	
A:102:_:ASN	HBOND:MC_MC	A:106:_:PHE	2.864	9.245	17	O	N	A:106:_:PHE	
A:103:_:ILE	HBOND:MC_MC	A:106:_:PHE	3.298	59.195	17	O	N	A:106:_:PHE	
A:103:_:ILE	HBOND:MC_MC	A:107:_:PHE	3.169	8.548	17	O	N	A:107:_:PHE	
A:103:_:ILE	VDW:SC_SC	A:107:_:PHE	3.653	-999.9	6	CG2	CE2		
A:103:_:ILE	VDW:SC_SC	A:128:_:PHE	3.993	-999.9	6	CG2	CE1		
A:104:_:GLU	HBOND:MC_MC	A:107:_:PHE	3.352	55.752	17	O	N	A:107:_:PHE	
A:104:_:GLU	HBOND:MC_MC	A:108:_:LEU	2.973	6.751	17	O	N	A:108:_:LEU	
A:104:_:GLU	VDW:MC_SC	A:108:_:LEU	4.024	-999.9	6	C	CD1		
A:104:_:GLU	VDW:SC_SC	A:115:_:PHE	3.93	-999.9	6	CB	CE1		
A:105:_:ALA	HBOND:MC_MC	A:108:_:LEU	3.41	59.24	17	O	N	A:108:_:LEU	
A:105:_:ALA	HBOND:MC_MC	A:109:_:LYS	2.901	13.414	17	O	N	A:109:_:LYS	
A:106:_:PHE	HBOND:MC_MC	A:109:_:LYS	3.343	49.773	17	O	N	A:109:_:LYS	
A:106:_:PHE	HBOND:MC_MC	A:110:_:MET	3.343	25.562	17	O	N	A:110:_:MET	
A:106:_:PHE	VDW:SC_SC	A:127:_:PHE	3.491	-999.9	6	CE1	CB		
A:106:_:PHE	PIPISTACK:SC_SC	A:128:_:PHE	6.05	150.937	9.4	7.086,-10.191,45.301	5.193,-5.719,41.692		P n1.00,p7.46
A:106:_:PHE	VDW:SC_SC	A:128:_:PHE	3.719	-999.9	6	CE2	CB		
A:106:_:PHE	VDW:SC_SC	A:131:_:GLU	3.837	-999.9	6	CG	CG		
A:107:_:PHE	HBOND:MC_MC	A:110:_:MET	3.369	37.243	17	O	N	A:110:_:MET	
A:107:_:PHE	HBOND:MC_MC	A:111:_:GLY	3.019	42.523	17	O	N	A:111:_:GLY	
A:107:_:PHE	HBOND:MC_MC	A:112:_:GLU	2.879	16.544	17	O	N	A:112:_:GLU	
A:107:_:PHE	VDW:SC_SC	A:112:_:GLU	3.866	-999.9	6	CD1	CB		
A:107:_:PHE	PIPISTACK:SC_SC	A:115:_:PHE	5.86	102.791	9.4	10.240,-7.212,42.593	15.224,-4.686,44.360		L n4.31,p4.28
A:107:_:PHE	VDW:SC_SC	A:115:_:PHE	3.5	-999.9	6	CB	CD1		
A:107:_:PHE	VDW:SC_MC	A:116:_:ASN	3.975	-999.9	6	CE1	C		
A:107:_:PHE	VDW:SC_SC	A:120:_:LEU	3.876	-999.9	6	CE1	CD1		
A:107:_:PHE	PIPISTACK:SC_SC	A:128:_:PHE	5.34	131.516	9.4	10.240,-7.212,42.593	5.193,-5.719,41.692		T-EF n2.63,p4.15
A:107:_:PHE	VDW:SC_SC	A:128:_:PHE	3.675	-999.9	6	CE2	CE1		
A:108:_:LEU	HBOND:MC_MC	A:111:_:GLY	3.327	29.6	17	O	N	A:111:_:GLY	
A:108:_:LEU	VDW:SC_SC	A:113:_:SER	3.868	-999.9	6	CD2	CB		
A:108:_:LEU	VDW:SC_SC	A:115:_:PHE	4.007	-999.9	6	CG	CE1		
A:110:_:MET	VDW:SC_SC	A:120:_:LEU	3.525	-999.9	6	CE	CD2		
A:110:_:MET	VDW:SC_SC	A:124:_:ASN	3.338	-999.9	6	CE	ND2		
A:112:_:GLU	HBOND:MC_MC	A:115:_:PHE	3.336	10.04	17	O	N	A:115:_:PHE	
A:112:_:GLU	VDW:SC_SC	A:119:_:LEU	3.795	-999.9	6	CD	CB		
A:116:_:ASN	HBOND:MC_MC	A:119:_:LEU	3.189	9.967	17	O	N	A:119:_:LEU	
A:117:_:PRO	HBOND:MC_MC	A:120:_:LEU	3.196	15.557	17	O	N	A:120:_:LEU	
A:117:_:PRO	VDW:SC_SC	A:128:_:PHE	3.534	-999.9	6	CG	CE2		
A:120:_:LEU	VDW:SC_SC	A:124:_:ASN	3.994	-999.9	6	CD2	CB		
A:120:_:LEU	VDW:SC_SC	A:128:_:PHE	3.595	-999.9	6	CD1	CB		
A:121:_:ASN	HBOND:SC_MC	A:124:_:ASN	2.93	19.856	17	OD1	N	A:124:_:ASN	
A:121:_:ASN	VDW:SC_SC	A:124:_:ASN	3.369	-999.9	6	OD1	CB		
A:121:_:ASN	HBOND:MC_MC	A:125:_:LEU	2.848	13.448	17	O	N	A:125:_:LEU	
A:122:_:GLN	HBOND:MC_MC	A:126:_:LYS	3.371	16.204	17	O	N	A:126:_:LYS	
A:123:_:GLN	HBOND:MC_MC	A:126:_:LYS	3.216	50.655	17	O	N	A:126:_:LYS	
A:123:_:GLN	HBOND:MC_MC	A:127:_:PHE	3.081	11.642	17	O	N	A:127:_:PHE	
A:123:_:GLN	VDW:SC_SC	A:127:_:PHE	3.286	-999.9	6	NE2	CZ		
A:124:_:ASN	HBOND:MC_MC	A:127:_:PHE	3.402	58.886	17	O	N	A:127:_:PHE	
A:124:_:ASN	VDW:SC_SC	A:127:_:PHE	3.555	-999.9	6	OD1	CD2		
A:124:_:ASN	HBOND:MC_MC	A:128:_:PHE	2.856	15.795	17	O	N	A:128:_:PHE	
A:125:_:LEU	HBOND:MC_MC	A:128:_:PHE	3.375	55.728	17	O	N	A:128:_:PHE	
A:125:_:LEU	HBOND:MC_MC	A:129:_:LEU	2.966	12.655	17	O	N	A:129:_:LEU	
A:126:_:LYS	HBOND:MC_MC	A:129:_:LEU	3.373	55.501	17	O	N	A:129:_:LEU	
A:126:_:LYS	HBOND:MC_MC	A:130:_:ASN	3.097	16.813	17	O	N	A:130:_:ASN	
A:127:_:PHE	HBOND:MC_MC	A:130:_:ASN	3.27	52.513	17	O	N	A:130:_:ASN	
A:127:_:PHE	HBOND:MC_MC	A:131:_:GLU	3.186	15.123	17	O	N	A:131:_:GLU	
A:128:_:PHE	HBOND:MC_MC	A:131:_:GLU	3.154	51.898	17	O	N	A:131:_:GLU	
A:128:_:PHE	HBOND:MC_MC	A:132:_:LEU	3.032	10.824	17	O	N	A:132:_:LEU	
A:128:_:PHE	VDW:SC_SC	A:132:_:LEU	3.708	-999.9	6	CE2	CD1		
A:129:_:LEU	HBOND:MC_MC	A:132:_:LEU	3.329	57.273	17	O	N	A:132:_:LEU	
A:129:_:LEU	HBOND:MC_MC	A:133:_:LEU	2.865	17.656	17	O	N	A:133:_:LEU	
A:130:_:ASN	HBOND:MC_MC	A:133:_:LEU	3.227	51.184	17	O	N	A:133:_:LEU	
A:130:_:ASN	HBOND:MC_MC	A:134:_:ILE	2.971	16.447	17	O	N	A:134:_:ILE	
A:131:_:GLU	HBOND:MC_MC	A:134:_:ILE	3.422	48.634	17	O	N	A:134:_:ILE	
A:131:_:GLU	HBOND:MC_MC	A:135:_:ALA	2.852	25.53	17	O	N	A:135:_:ALA	
**LIGAND INTERACTIONS**									
_:1:_:FMN	IAC:LIG_SC	A:7:_:TYR	2.968	-999.9	0	C6	HH		
_:1:_:FMN	IAC:LIG_SC	A:9:_:SER	4.789	-999.9	0	C7M	OG		
_:1:_:FMN	IAC:LIG_SC	A:11:_:ARG	6.956	-999.9	0	C8M	HA		
_:1:_:FMN	IAC:LIG_SC	A:21:_:ASP	6.488	-999.9	0	C8M	HB3		
_:1:_:FMN	IAC:LIG_SC	A:22:_:LEU	6.388	-999.9	0	C7M	HD22		
_:1:_:FMN	IAC:LIG_MC	A:24:_:ASP	6.466	-999.9	0	C1'	O		
_:1:_:FMN	VDW:LIG_SC	A:25:_:ILE	3.745	-999.9	6	C6	CG2		
_:1:_:FMN	IAC:LIG_SC	A:26:_:LEU	6.235	-999.9	0	N5	HD22		
_:1:_:FMN	IAC:LIG_SC	A:28:_:GLU	2.551	-999.9	0	O3'	OE1		
_:1:_:FMN	VDW:LIG_SC	A:29:_:ALA	3.299	-999.9	6	C4	CB		
_:1:_:FMN	IAC:LIG_MC	A:30:_:ARG	5.472	-999.9	0	N3	N		
_:1:_:FMN	IAC:LIG_MC	A:31:_:ASP	6.185	-999.9	0	O2	C		
_:1:_:FMN	IAC:LIG_SC	A:32:_:PHE	2.296	-999.9	0	O2	HB3		
_:1:_:FMN	VDW:LIG_SC	A:33:_:ASN	3.673	-999.9	6	C4	OD1		
_:1:_:FMN	IAC:LIG_SC	A:36:_:ASN	5.922	-999.9	0	O2	HD21		
_:1:_:FMN	IAC:LIG_SC	A:38:_:ILE	4.501	-999.9	0	O4	HG21		
_:1:_:FMN	IAC:LIG_MC	A:39:_:CYS	6.747	-999.9	0	O4	O		
_:1:_:FMN	IAC:LIG_SC	A:40:_:GLY	6.623	-999.9	0	O4	HA1		

_:1:_:FMN	IAC:LIG_SC	A:42:_:LEU	3.218	-999.9	0	C7M	HD11
_:1:_:FMN	IAC:LIG_MC	A:43:_:TYR	6.97	-999.9	0	C7M	O
_:1:_:FMN	IAC:LIG_SC	A:44:_:TYR	6.44	-999.9	0	C7M	HD1
_:1:_:FMN	IAC:LIG_SC	A:49:_:PHE	3.689	-999.9	0	C7M	CG
_:1:_:FMN	IAC:LIG_MC	A:50:_:PHE	6.596	-999.9	0	C7M	N
_:1:_:FMN	VDW:LIG_SC	A:51:_:GLN	3.407	-999.9	6	C6	OE1
_:1:_:FMN	IAC:LIG_MC	A:52:_:CYS	6.477	-999.9	0	O4	O
_:1:_:FMN	IAC:LIG_SC	A:53:_:LEU	2.883	-999.9	0	O4	HD23
_:1:_:FMN	VDW:LIG_SC	A:63:_:LEU	3.454	-999.9	6	C2	CD1
_:1:_:FMN	IAC:LIG_SC	A:66:_:LYS	3.079	-999.9	0	O5'	HD2
_:1:_:FMN	IAC:LIG_SC	A:67:_:ILE	2.652	-999.9	0	O2'	HA
_:1:_:FMN	IAC:LIG_MC	A:68:_:GLN	5.792	-999.9	0	O2'	N
_:1:_:FMN	IAC:LIG_SC	A:69:_:LYS	4.92	-999.9	0	O4'	HB2
_:1:_:FMN	IAC:LIG_SC	A:70:_:ASP	2.657	-999.9	0	O2'	HB2
_:1:_:FMN	IAC:LIG_SC	A:71:_:GLN	5.599	-999.9	0	O4'	H
_:1:_:FMN	IAC:LIG_SC	A:72:_:ARG	3.091	-999.9	0	C1'	HH21
_:1:_:FMN	IAC:LIG_SC	A:73:_:HIS	3.659	-999.9	0	C7M	ND1
_:1:_:FMN	IAC:LIG_MC	A:93:_:SER	5.439	-999.9	0	O4	O
_:1:_:FMN	IAC:LIG_SC	A:94:_:MET	2.874	-999.9	0	O4	HE1
